# Effects of intravenous AICAR (5-aminoimidazole-4-carboximide riboside) administration on insulin signaling and resistance in premature baboons, *Papio sp*.

**DOI:** 10.1371/journal.pone.0208757

**Published:** 2018-12-12

**Authors:** Cynthia L. Blanco, Amalia Gastaldelli, Diana G. Anzueto, Lauryn A. Winter, Steven R. Seidner, Donald C. McCurnin, Hanyu Liang, Martin A. Javors, Ralph A. DeFronzo, Nicolas Musi

**Affiliations:** 1 Department of Pediatrics, Division of Neonatology, University of Texas Health Science Center at San Antonio, San Antonio, TX, United States of America; 2 Department of Medicine, Division of Diabetes, University of Texas Health Science Center at San Antonio, San Antonio, TX, United States of America; 3 Institute of Clinical Physiology Consiglio Nazionale delle Ricerche, Pisa Italy; 4 Department of Psychiatry, University of Texas Health Science Center at San Antonio, San Antonio, TX, United States of America; 5 Department of Pharmacology, University of Texas Health Science Center at San Antonio, San Antonio, TX, United States of America; 6 Texas Diabetes Institute, San Antonio, TX, United States of America; 7 Sam and Ann Barshop Institute for Longevity and Aging Studies, San Antonio, TX, United States of America; Medical University of Vienna, AUSTRIA

## Abstract

Premature baboons exhibit peripheral insulin resistance and impaired insulin signaling. 5’ AMP-activated protein kinase (AMPK) activation improves insulin sensitivity by enhancing glucose uptake (via increased glucose transporter type 4 [GLUT4] translocation and activation of the extracellular signal-regulated kinase [ERK]/ atypical protein kinase C [aPKC] pathway), and increasing fatty acid oxidation (via inhibition of acetyl-CoA carboxylase 1 [ACC]), while downregulating gluconeogenesis (via induction of small heterodimer partner [SHP] and subsequent downregulation of the gluconeogenic enzymes: phosphoenolpyruvate carboxykinase [PEPCK], glucose 6-phosphatase [G6PASE], fructose- 1,6-bisphosphatase 1 [FBP1], and forkhead box protein 1 [FOXO1]). The purpose of this study was to investigate whether pharmacologic activation of AMPK with AICAR (5-aminoimidazole-4-carboximide riboside) administration improves peripheral insulin sensitivity in preterm baboons. 11 baboons were delivered prematurely at 125±2 days (67%) gestation. 5 animals were randomized to receive 5 days of continuous AICAR infusion at a dose of 0.5 mg·g^-1^·day^-1^. 6 animals were in the placebo group. Euglycemic hyperinsulinemic clamps were performed at 5±2 and 14±2 days of life. Key molecules potentially altered by AICAR (AMPK, GLUT4, ACC, PEPCK, G6PASE, FBP1, and FOXO1), and the insulin signaling molecules: insulin receptor (INSR), insulin receptor substrate 1 (IRS-1), protein kinase B (AKT), and peroxisome proliferator-activated receptor gamma coactivator 1-alpha (PGC1α) were measured using RT-PCR and western blotting. AICAR infusion did not improve whole body insulin-stimulated glucose disposal in preterm baboons (12.8±2.4 vs 12.4±2.0 mg/(kg·min), p = 0.8, placebo vs AICAR). One animal developed complications during treatment. In skeletal muscle, AICAR infusion did not increase phosphorylation of ACC, AKT, or AMPK whereas it increased mRNA expression of *ACACA* (ACC), *AKT*, and *PPARGC1A* (PGC1α). In the liver, *INSR*, *IRS1*, *G6PC3*, *AKT*, *PCK1*, *FOXO1*, and *FBP1* were unchanged, whereas *PPARGC1A* mRNA expression increased after AICAR infusion. This study provides evidence that AICAR does not improve insulin sensitivity in premature euglycemic baboons, and may have adverse effects.

## Introduction

Prematurity is a major cause of morbidity and mortality among neonates, including a higher risk of developing type 1 and 2 diabetes earlier in life [1–3]. Both extremely premature human neonates [4] and extremely premature baboon neonates [5] exhibit postnatal insulin resistance, contributing to the development of hyperglycemia and its related morbidities [6–8]. The etiology of insulin resistance in preterm infants is not well-understood; however, we have previously demonstrated in premature baboons that impaired insulin signaling in the skeletal muscle and liver, and impaired suppression of hepatic gluconeogenesis likely contribute [5,9,10]. In the insulin signaling cascade, insulin binding activates the insulin receptor (INSR), which in turn activates insulin receptor substrate adapters including insulin receptor substrate 1 (IRS1). Phosphorylated IRS1 interacts with signaling molecules including phosphatidylinositol-4,5-bisphosphate 3-kinases (PI3Ks), which then activate the protein kinase B (AKT) cascade [11]. AKT activation promotes glucose uptake through increased glucose transporter type 4 (GLUT4, encoded by *SLC2A4*) translocation, which leads to increased glucose uptake in muscle and adipose tissues. AKT activation also increases glucose storage as glycogen through inhibition of glycogen synthase kinase 3 (GSK3)[12]. Finally, the transcriptional coactivator, peroxisome proliferator-activated receptor gamma coactivator 1-alpha (PGC1α) also plays a key role in coordinating cellular metabolic pathways and insulin sensitivity [13].

5’ AMP-activated protein kinase (AMPK) is another key metabolic regulatory protein. AMPK activation stimulates fatty acid oxidation through phosphorylation of acetyl-CoA carboxylase 1 (ACC, encoded by *ACACA*)[14] and downregulates lipolysis, likely through regulation of hormone-sensitive lipase (HSL) and adipose triglyceride lipase (ATGL) [15]; thereby resulting in reduced serum free fatty acids (FFA). AMPK also inhibits gluconeogenesis through activation of the orphan nuclear receptor small heterodimer partner (SHP) [16], which in turn downregulates key enzymes involved in gluconeogenesis, including: phosphoenolpyruvate carboxykinase (PEPCK, encoded by *PCK1*), glucose 6-phosphatase (G6PASE, encoded by *G6PC3*), fructose- 1,6-bisphosphatase 1 (FBPASE, encoded by *FBP1*), and forkhead box protein 1 (FOXO1, encoded by *FOXO1*) [17]. Finally, AMPK augments glucose uptake in skeletal muscle through a PI 3-kinase independent pathway in association with enhanced GLUT4 translocation [18,19], likely via TBC1 domain family member 4 (TBC1D4) signaling [20,21]. Therefore, stimulation of AMPK by pharmacological methods may represent a novel therapeutic approach for improving peripheral insulin resistance in premature neonates.

AICAR (5-aminoimidazole-4-carboximide riboside) is an adenosine analog that can be phosphorylated to form AICAR monophosphate (ZMP). ZMP then activates AMPK [22]. ZMP has also been demonstrated to inhibit hepatic gluconeogenesis independently of AMPK activation [23]. Independent of AMPK activation, AICAR also stimulates glucose uptake via the extracellular signal-regulated kinase (ERK)/ atypical protein kinase C (aPKC) pathway [24]. These properties make AICAR a potential candidate to improve insulin resistance in premature infants, and several studies have shown that AICAR can reduce hepatic gluconeogenesis and improve glucose uptake in adults [25,26].

The baboon, *Papio sp*., is an excellent animal model to study the insulin resistance of prematurity because baboons are extremely close to humans phylogenetically, with estimated 97% sequence conservation. Similar to humans, preterm baboons have greater than 90% incidence of developing hyperglycemia spontaneously, have downregulation of GLUT4 shortly after birth, and exhibit decreased peripheral insulin sensitivity two weeks after birth [5,7,27]. Finally, baboons spontaneously develop insulin resistance and diabetes later in life [10,28].

The purpose of this study was to determine if pharmacologic activation of AMPK by administration of AICAR improves peripheral insulin resistance in preterm baboons by upregulating skeletal muscle *SLC2A4* gene expression and GLUT4 protein content or upregulation of other key insulin signaling molecules.

## Materials and methods

### Animal care

All animal experiments were approved by the Institutional Animal Care and Use Committee (Protocol Number: 20110081AR (11081X)) at the University of Texas Health Science Center (San Antonio, Texas, USA) and were conducted in accordance with accepted standards of humane animal use. All efforts were made to minimize suffering. A total of 11 preterm baboons were studied, with five animals in the treatment group and six animals in the placebo group. Animals (*Papio anubis* and *Papio cynocephalus* x *Papio anubis* hybrids) were obtained from the Southwest National Primate Center at the Texas Biomedical Research Institute (San Antonio, TX, USA). Studies were performed at the University of Texas Health Science Center. Animals were delivered at 125±2 days (67%) gestation via cesarean section under general anesthesia from healthy, non-diabetic mothers.

#### Care of preterm animals

Routine care of preterm animals was performed as previously described [5]. Immediately after delivery, animals were intubated, administered Surfactant (Survanta, 4ml/kg, Abbott Laboratories, Abbott Park, USA), and placed on mechanical ventilation (VIP Gold Bird, CareFusion, San Diego, USA). Arterial blood gases were monitored (at minimum) every 2–4 hours using an I-Stat handheld blood gas monitoring system (Abbott). Ventilator settings were adjusted based on blood gas results according to a standardized protocol. An umbilical arterial catheter and peripherally inserted central venous catheter (PICC) were placed to establish arterial and central venous access, respectively. Ketamine (2mg, Putney, Portland, USA) and Midazolam (0.02mg, Akorn, Lake Forest, USA) were administered every 2–6 hours for sedation and analgesia. Shortly after birth, 2.5% intravenous dextrose was initiated and glucose infusion rate (GIR) was titrated to maintain target glucose of 50–100 mg/dL. Plasma glucose was measured after birth and at least every 4 hours thereafter. Urine glucose was measured at least every 12 hours. Intravenous insulin was not administered for medical treatment. Total parenteral nutrition was initiated on day of life (DOL) 0 with 5% dextrose solution at a rate of 150 ml·kg^-1^·day^-1^. GIR was increased every 1–2 days as tolerated to insure adequate caloric intake. Amino acids (Trophamine, B. Braun, Bethlehem, USA) were added to the parenteral nutrition solution on DOL 1 at a rate of 1.75 g·kg^-1^·day^-1^and increased on DOL 2 to 3.5 g·kg^-1^·day^-1^. Intravenous lipids were not administered. Animals were housed in Dräger Caleo infant incubators (Drägerwerk AG & Co., Lübeck, Germany). Nursing care was provided 24 hours/day for the duration of the study. Intravenous nutrition and other infusions were administered via Alaris PC pump modules (BD, CareFusion), and Baxter AS50 syringe pumps (Baxter, Deerfield, USA). Vital signs were monitored via Datascope Passport XG monitoring systems (Datascope Corp. Fairfield, USA). Animals were euthanized with exsanguination followed by pentobarbital.

#### AICAR administration

Animals were randomly assigned to the placebo or AICAR group by computerized randomization on DOL5±2. Syringes were prepared daily by a research assistant not involved with clinical care of the baboons and investigators/ personnel providing direct care remained blinded to group allocation. Three animals received placebo and three other animals assigned to the placebo group received no placebo infusion to serve as shared controls for another study to minimize the number of non-human primates being euthanized, as requested by the IACUC. Five premature baboons received 5 days of AICAR (Toronto Research Chemicals, North York, Canada) infusion. AICAR infusion was administered as an initial prime or bolus dose, followed by a continuous (24hr) infusion, delivered at an appropriate cc/hr rate to achieve the correct mg of AICAR per day. Two animals in the AICAR treatment group initially received a prime dose (0.1 mg/g body weight) of AICAR followed by a continuous infusion at 0.5 mg·g^-1^·day^-1^ for 5 days, as described by Cuthbertson [26]. However, premature animals that received this dose had a lower than expected serum AICAR concentration. Therefore, in the third premature baboon, the dose of AICAR was increased to a prime of 0.15 mg/g followed by a continuous infusion of 1.5 mg·g^-1^·day^-1^ for 5 days. However, this animal had a serum AICAR concentration that reached toxic levels and developed complications (see results/discussion). Therefore, in the last two animals, the AICAR prime was reduced to 0.15 mg/g followed by a continuous infusion of 0.5 mg·g^-1^·day^-1^(i.e., similar to the first two treated animals) for 5 days.

#### Euglycemic hyperinsulinemic clamp procedure

Insulin sensitivity was measured by performing euglycemic hyperinsulinemic clamps as previously described [5,29]. Insulin clamps were performed on day 5±2 of life and again on day 14±2 of life. Parenteral nutrition was discontinued 12 hours prior to each insulin clamp study. Each insulin clamp lasted 120 minutes. Animals received a prime (150 mU/kg, given over 1 minute) plus constant infusion of insulin at a rate of 15 mU·kg^-1^·min^-1^ (Novolin; Novo Nordisk Pharmaceuticals, Princeton, USA). Simultaneously, glucose (25% dextrose in water, Hospira, USA) was infused at a variable rate to maintain the blood glucose concentration at 60–80 mg/dL. Blood glucose was measured every 5–10 minutes during the insulin clamp in order to appropriately adjust the GIR to maintain euglycemia. Plasma samples were collected at -180, 0, +30, +60, +90, and +120 min for determination of insulin concentration (Ultrasensitive Insulin ELISA kit, ALPCO Diagnostics, Salem, USA). Insulin sensitivity was calculated as previously described [5,29]. Briefly, the value of *M*, or glucose metabolized [expressed as mg/(kg·min)], was calculated according to the equation:

M=GIR−SC

Where GIR is the glucose infusion rate and SC is the space correction (to correct for instability of plasma glucose concentrations). The space correction was calculated based on the following equation:

$$SC=\left(G_{2}-G_{1}\right)\cdot 0.095$$

Where: G_2_ and G_1_ are the blood glucose concentrations in mg/dL at the end and at the beginning of the time interval, respectively.

The total *M* for each insulin clamp study was calculated from the means of the five 20 minute study intervals between 20 and 120 minutes of the insulin clamp.

Plasma glucagon (EIA Kit, ALPCO Diagnostics, Salem, USA), plasma epinephrine (2-CAT [A-N] Research ELISA Enzyme Immunoassay, Labor Diagnostika Nord GMBH & Co., Nordhorn, Germany), and serum free fatty acids (FFA) (HR Series NEFA-HR Kit, Wako Diagnostics, Mountain View, USA) were measured at time 0 and at 120 minutes. No animals had elevated levels of any counter-regulatory hormones at the end of the insulin clamp compared to the basal level. Serum AICAR concentration was measured by mass spectrometry [30] before AICAR administration, daily during AICAR infusion, and at necropsy. Animals were normotensive, euglycemic, and off all medications that could influence glucose metabolism for >24 hours prior to the insulin clamp.

#### Tissue collection

Two muscle biopsies were performed during each insulin clamp (one taken just prior to the start of the clamp, and the second taken at the end of the clamp procedure). Muscle biopsies were obtained from the biceps femoris muscle via sharp dissection using sterile technique from one leg for the first biopsy and from the contralateral leg for the second biopsy. Animals were anesthetized and received 1% lidocaine locally prior to the biopsies to obtain ~10 mg of muscle tissue. The muscle tissue was snap-frozen in liquid nitrogen and stored at -80°C. Liver tissue was collected immediately after euthanasia, snap frozen and stored as above.

### Glycogen content

Glycogen content was measured in liver and skeletal muscle samples obtained at necropsy as previously described [9].

### Measurement of insulin signaling and glucose transport proteins

Archived protocols for western blotting and PCR can be found at DOIs: dx.doi.org/10.17504/protocols.io.tpqemmw and dx.doi.org/10.17504/protocols.io.tpsemne, respectively.

#### Western blot

Key insulin signaling (ACC, AKT, AMPK, ERK, FOXO1, FBPASE) and glucose transport (GLUT4) proteins were measured from muscle and/or liver tissue as previously described [10]. To verify these results, and ensure that the buffers were not interacting with AICAR directly, we repeated all western blots using two different buffers: Buffer A (10 μg/μL aprorotinin, 10 μg/mL, leupeptin 10 μg/mL, benzamidine 3 mmol/L, PMSF 1 mmol/L, 1% (v/v) nonidet P-40, EDTA 5 mmol/L (pH 8.0), sodium orthovanadate 2 mmol/L, sodium fluoride 100 mmol/L, sodium pyrophosphate 10 mmol/L, and Tris 20 mmol/L (pH 7.5)) and Buffer B (20 mmol/L Tris, 50 mmol/L NaCl, 50 mmol/L NaF, 5 mmol/L sodium pyrophosphate, 250 mmol/L sucrose, 1% (v/v) Triton-X, 2 μmol/L DTT, 0.05 mg/mL soyabean trypisn inhibitor, 0.004 mg/mL leupeptin, 0.099 mmol/L benzamidine, 0.5 mmol/L PMSF). Reagents were purchased from Sigma-Aldrich (St. Louis, USA), Thermo-Fisher (Waltham, USA), and/or BioRad Laboratories (Hercules, USA). Antibodies used are summarized in Table 1. Band intensity was quantified by densitometry using ImageJ (National Institutes of Health, Bethesda, USA) and results reported in arbitrary OD units. Glyceraldehyde-3-phosphate dehydrogenase (GAPDH) was used as a loading control. Gels were normalized using appropriate internal controls and phosphorylated proteins were normalized to their total protein content. Raw images of western blot membranes used in analysis can be found in S1 Images.

**Table 1:** Summary of western blot antibodies.

Target	Manufacturer information	Host species	Clonality	Dilution used
**ACC**	Cell Signaling, #3676, Beverly, MA 01915	Rabbit	Monoclonal	1:200; 1:500
**pACC**	Cell Signaling, #11818, Beverly, MA 01915	Rabbit	Monoclonal	1:100; 1:500
**ERK**	Millipore, 06–182, Billerica, MA 01821	Rabbit	Polyclonal	1:1000; 1:1200
**pERK**	Millipore, 05-797R, Billerica, MA 01821	Rabbit	Monoclonal	1:200; 1:500; 1:1000
**AKT**	Cell Signaling, #9272, Beverly, MA 01915	Rabbit	Polyclonal	1:500; 1:1000
**pAKT**	Cell Signaling, #9271, Beverly, MA 01915	Rabbit	Polyclonal	1:100; 1:200; 1:500
**GLUT4**	Abcam, ab654, Cambridge, MA 02139	Rabbit	Polyclonal	1:800; 1:1000
**AMPK**	Cell Signaling, #2532, Beverly, MA 01915	Rabbit	Polyclonal	1:100; 1:200
**pAMPK**	Cell Signaling, #2535, Beverly, MA 01915	Rabbit	Monoclonal	1:100
**GAPDH**	Santa Cruz Biotechnology, sc-25778, Dallas, TX 75220	Rabbit	Polyclonal	1:1000
**FBPASE**	Santa Cruz Biotechnology, sc-66946, Dallas, TX 75220	Rabbit	Polyclonal	1:1000
**FOXO1**	Santa Cruz Biotechnology, sc-67140, Dallas, TX 75220	Rabbit	Polyclonal	1:200
**pFOXO1**	Santa Cruz Biotechnology, sc-101681, Dallas, TX 75220	Rabbit	Polyclonal	1:200
**Rabbit IgG HRP (secondary antibody)**	GE Healthcare UK Limited; NA934V, Buckinghamshire HP7 9NA, UK	Donkey	Polyclonal	1:1000; 1:1200; 1:1500

Summary of antibodies used for western blotting, including manufacturer information, host species, clonality, and dilutions used. A lower-case “p” before the gene name indicates the phosphorylated state.

#### PCR

Total RNA was extracted from muscle and liver tissue using the RNeasy mini kit from QIAGEN (USA). RT-PCR was performed as previously described, using TaqMan (Life Technologies, Carlsbad, USA) primers (summarized in Table 2) [31]. Relative quantitation of gene expression was accomplished using the relative standard curve method. The quantity of mRNA for each gene was normalized to importin 8 (*IPO8*) expression.

**Table 2:** Summary of RT-PCR primers.

Target gene	Species	Amplicon length	Manufacturer information
**ACACA**	Human	65	Life Technologies, Hs01046047_m1
**AKT1**	Human	66	Life Technologies, Hs00178289_m1
**PRKAA2**	Human	102	Life Technologies, Hs00178903_m1
**FOXO1**	Human	90	Life Technologies, Hs01054576_m1
**G6PC3**	Human	59	Life Technologies, Hs00292720_m1
**PPARGC1A**	Human	74	Life Technologies, Hs01016719_m1
**INSR**	Human	68	Life Technologies, Hs00961554_m1
**IRS1**	Human	69	Life Technologies, Hs00178563_m1
**PCK1**	Human	81	Life Technologies, Hs00159918_m1
**FBP1**	Human	80	Life Technologies, Hs00983323_m1
**IPO8**	Human	71	Life Technologies, Hs00183533_m1

Summary of primers used for RT-PCR, including species, amplicon length, and manufacturer information.

### Statistical analysis

Statistical calculations were performed with SPSS for Microsoft Windows, (Version 22.0, SPSS, Inc., Chicago, USA). Differences between groups were analyzed using independent-samples t-test for numerical data and chi-square for categorical data. *M* values were also compared from clamp 1 to clamp 2 by paired samples t-test. Results are reported as mean ± SD unless otherwise specified. The results of the insulin clamp studies are presented as follows: results from immediately prior to the start of the insulin clamp (time 0 minutes) are reported as “baseline” and results from the end of the insulin clamp (time 120 minutes) are reported as “insulin-stimulated”. A value of p<0.05 was considered significant. Sample size was determined as follows. The primary analysis was based on the difference of *M* value under insulin stimulation during the insulin clamp performed prior to AICAR infusion versus the insulin clamp performed after 5 days of AICAR infusion. Based on preliminary data where preterm baboons had lower insulin sensitivity than term baboons (preterm baboons had an *M* value of 17.6±2.7 and term baboons have an *M* value of 25.4±1.6), an improvement in insulin sensitivity in preterm animals to 90% of the *M* value of term baboons, with a 5% significance level and 80% power, a sample size of 4 animals per group was calculated. To account for expected death due to severity of illness of preterm baboons, we included 5–6 animals in each preterm group to achieve 4 completers per group.

## Results

All relevant raw data from this manuscript can be found archived at:DOI: 10.6084/m9.figshare.7392593.v1.

### Animal characteristics

**Fig 1:**
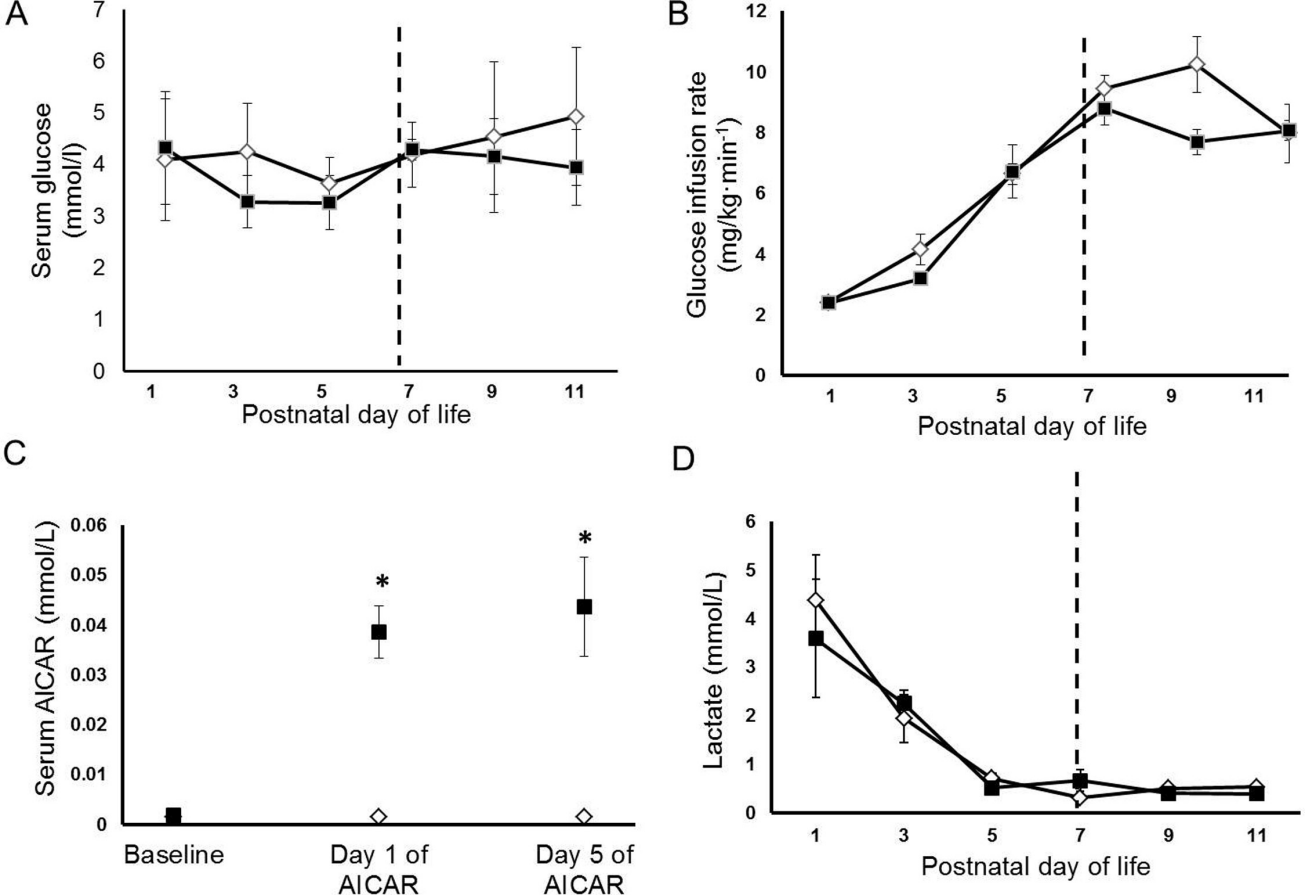
Serum values and glucose infusion rates in premature baboons. Average daily (A) serum glucose levels and (B) glucose infusion rates are shown for each group. (C) Serum levels of AICAR at baseline, day 1 of AICAR, and day 5 of AICAR infusion are shown for both groups. (D) Serum lactate levels for each treatment group are shown. Error bars represent ± standard error of the mean. Dashed line represents start of AICAR treatment. Black box represents the AICAR group, white diamond represents the placebo group.*, p<0.05, placebo vs AICAR, independent samples T-Test.

Six animals were allocated to the placebo group (mean birth weight: 381±50g, male/female: 3/3) and 5 animals to the AICAR group (mean birth weight: 382±33g, male/female: 3/2). No differences in weight or gender were present between groups (p = 0.97 and p = 0.74, respectively). Daily blood glucose levels and glucose infusion rates (to maintain euglycemia) were similar in placebo and AICAR treatment groups and are summarized in Fig 1 (A and B, respectively).

There were no early deaths during the course of the experiment. One animal in the AICAR group developed renal failure after 4 days of AICAR infusion as characterized by high blood urea nitrogen, hyperphosphatemia, and hyperkalemia (BUN: 34 mg/dL, Phos: 5 mg/dL, K: 7.2 meq/dL) and low sodium and total protein (Na = 133 meq/L, TP = 3.8 g/dL). Furthermore, this animal exhibited signs of stress, evidenced by high plasma epinephrine and glucagon concentrations, prior to the insulin clamp in addition to renal failure. Therefore, results of the insulin clamp and muscle biopsies after deterioration were excluded from analysis.

### Serum AICAR concentrations

Serum AICAR concentrations were similar in both groups at baseline (levels non-detectable) and remained undetectable in the placebo group (Fig 1C). Serum AICAR concentrations were significantly increased during AICAR infusion compared to placebo group on day 1 and day 5 (p = 0.02 and p = 0.02, respectively, Fig 1C).

### Effects of AICAR infusion

#### Plasma lactate and counter-regulatory hormones

**Fig 2:**
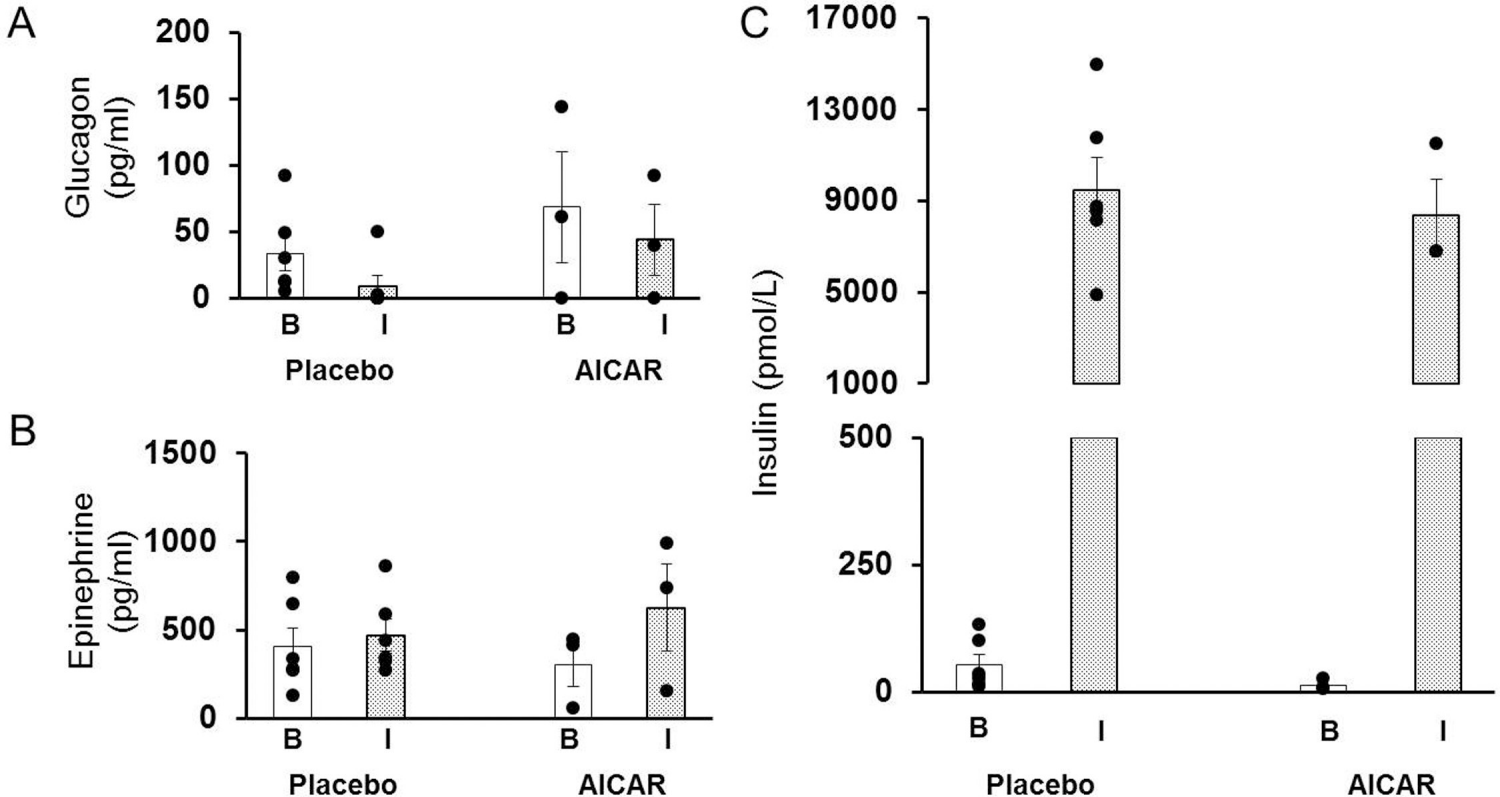
Counter-regulatory hormones and serum insulin concentrations in premature baboons at DOL 14. Plasma levels of (A) glucagon, and (B) epinephrine are shown for each treatment group at baseline (clamp time +0 minutes, B; white column) and under insulin stimulation (clamp time +120 minutes, I; dotted column). Serum insulin levels (C), expressed in pmol/L, at baseline (clamp time +0 minutes, B; white column) and under insulin stimulation (clamp time +120 minutes, I; dotted column) are shown for each treatment group. Break in the graph indicates where graph was superimposed to reduce the graph size. Error bars represent ± standard error of the mean.

Plasma lactate concentrations were measured daily as lactic acidosis has been described as a possible side effect of AICAR treatment [26]. No differences were seen between treatment groups (Fig 1D). As expected, serum lactate levels were elevated in both groups during the first few days of life after delivery, but decreased over time in both groups. Plasma glucagon and epinephrine did not vary significantly between treatment groups at the start of the insulin clamp (clamp time +0 minutes, hereafter “baseline” or “B”) or at the end of the insulin clamp studies (clamp time +120 minutes, hereafter “insulin simulated” or “I”; Fig 2A and 2B, respectively). These counter-regulatory hormones are within the normal fetal levels previously reported [5].

#### Insulin sensitivity

As intended, after each insulin clamp, the plasma insulin concentration was significantly increased to levels greater than 1000 pmol/L. This target was chosen to ensure that gluconeogenesis was completely suppressed in preterm baboons, as previously reported [5]. Neither baseline fasting serum insulin levels nor insulin-stimulated serum insulin levels were different between treatment groups at DOL 14 (p = 0.12 and p = 0.64, respectively, Fig 2C).

**Fig 3:**
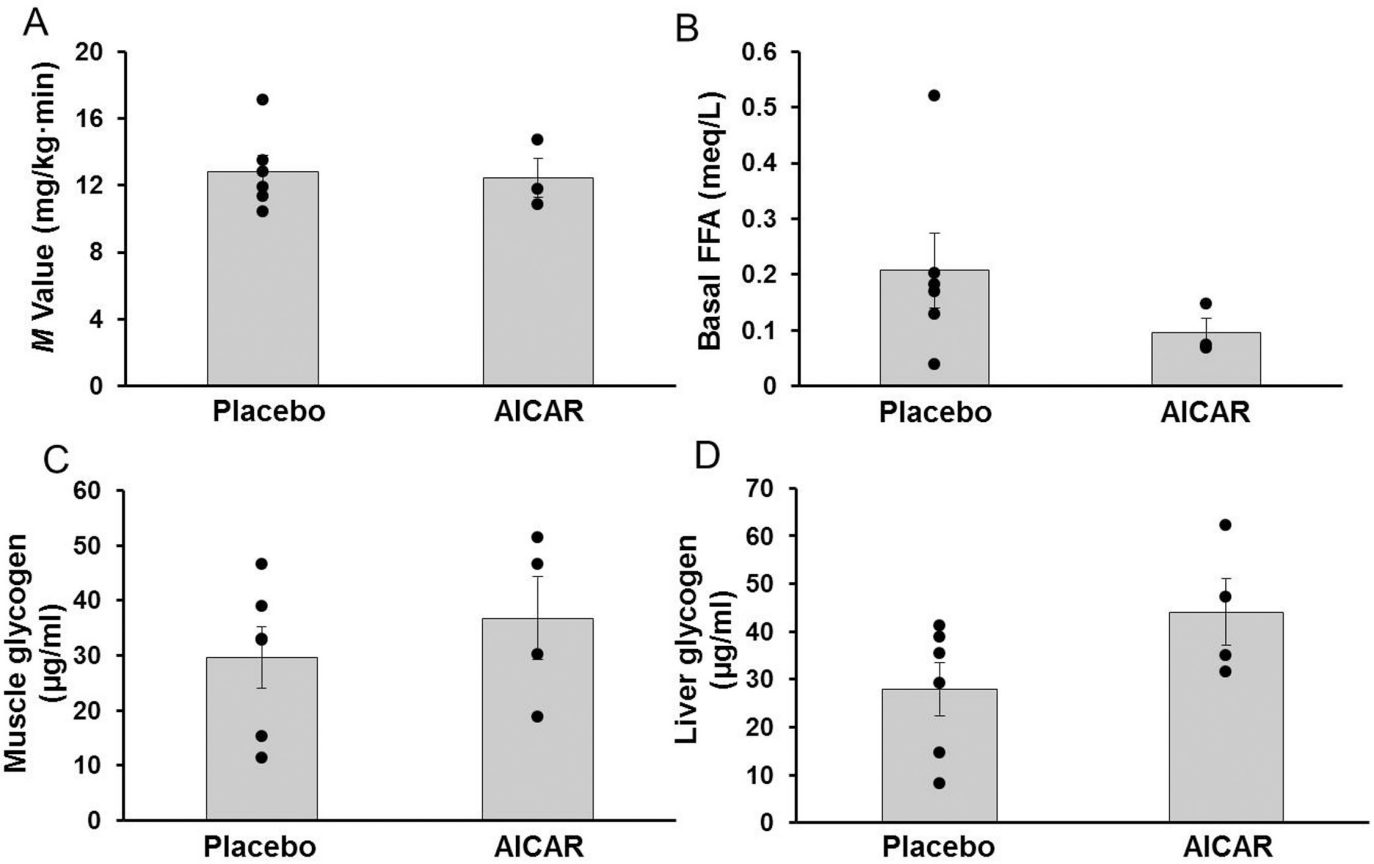
Insulin sensitivity, free fatty acids, and glycogen content in premature baboons on DOL 14. (A) Peripheral insulin sensitivity (*M*-value), and (B) serum levels of free fatty acids (FFA), are shown for placebo and AICAR treated animals. Glycogen content of (C) skeletal muscle and (D) liver for each treatment group is also shown. Error bars represent ± standard error of the mean.

Insulin sensitivity (*M*-value) at DOL 5 was similar between groups (*M*-value 12.8±1.7 vs 12.6±0.4 mg/(kg·min), p = 0.88, placebo vs AICAR prior to start of infusion, see archived data) and did not improve after 5 days of AICAR infusion (*M*-value 12.8±2.4 vs 12.4±2.0 mg/(kg·min) on DOL 14, p = 0.8, Fig 3A).

#### FFA

Baseline serum FFA was not significantly different between placebo treated and AICAR treated animals on DOL 14 (p = 0.3, Fig 3B).

#### Glycogen

Glycogen content in skeletal muscle and liver tissue at necropsy was similar between the preterm baboons treated with AICAR compared to the placebo animals (p = 0.46 and p = 0.11, respectively, Fig 3C and 3D, respectively).

#### Insulin signaling molecules in skeletal muscle

**Fig 4:**
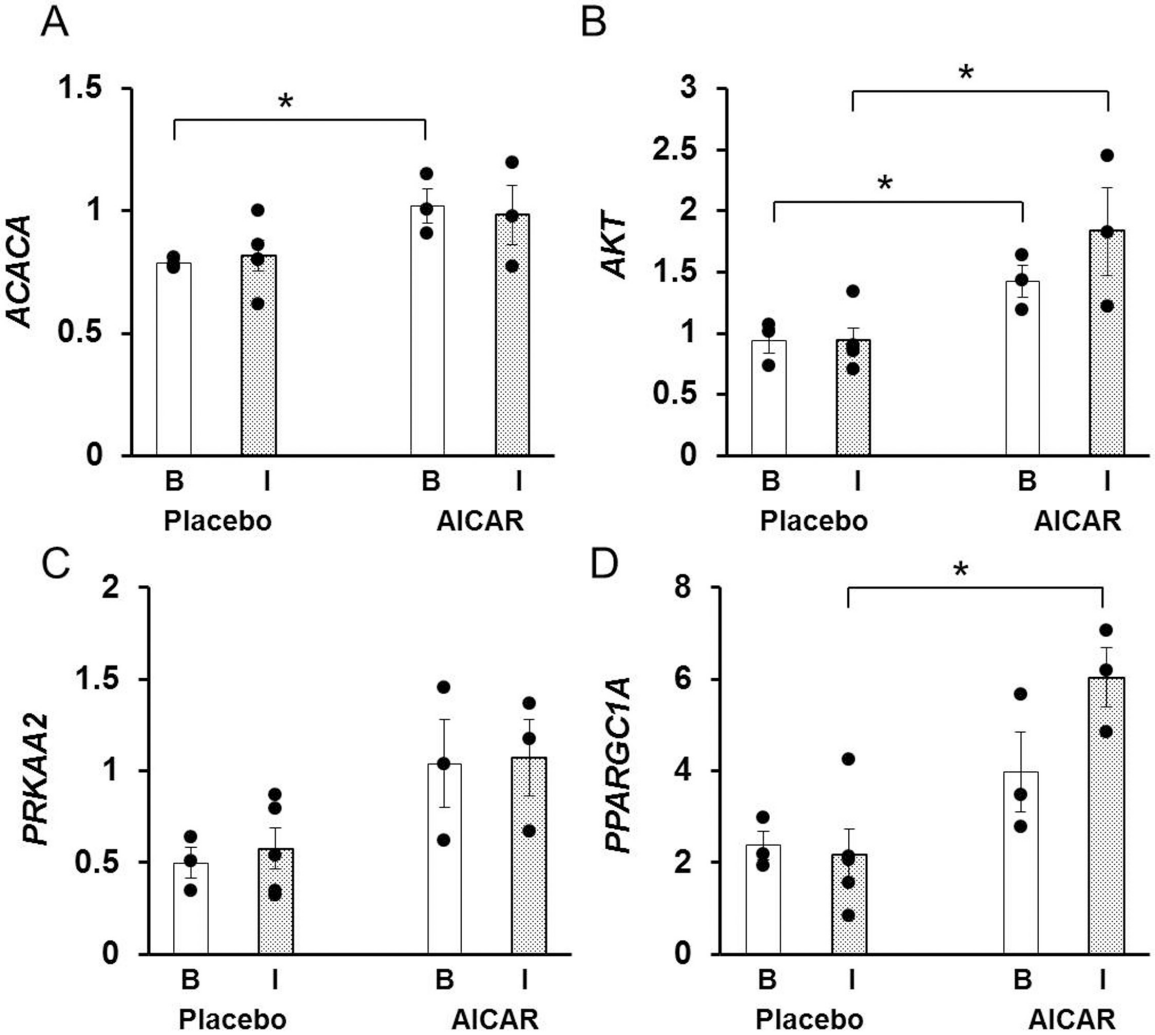
Skeletal muscle relative mRNA content of insulin signaling proteins in premature baboons on DOL 14. Relative mRNA content of (A) *ACACA*, (B) *AKT*, (C) *PRKAA2* and (D) *PPARGC1A* in skeletal muscle at baseline (clamp time +0 minutes, B; white column) and under insulin stimulation (clamp time +120 minutes, I; dotted column) is shown for both treatment groups. Error bars represent ± standard error of the mean. *, p< 0.05, placebo vs AICAR, independent samples T-Test.

**Fig 5:**
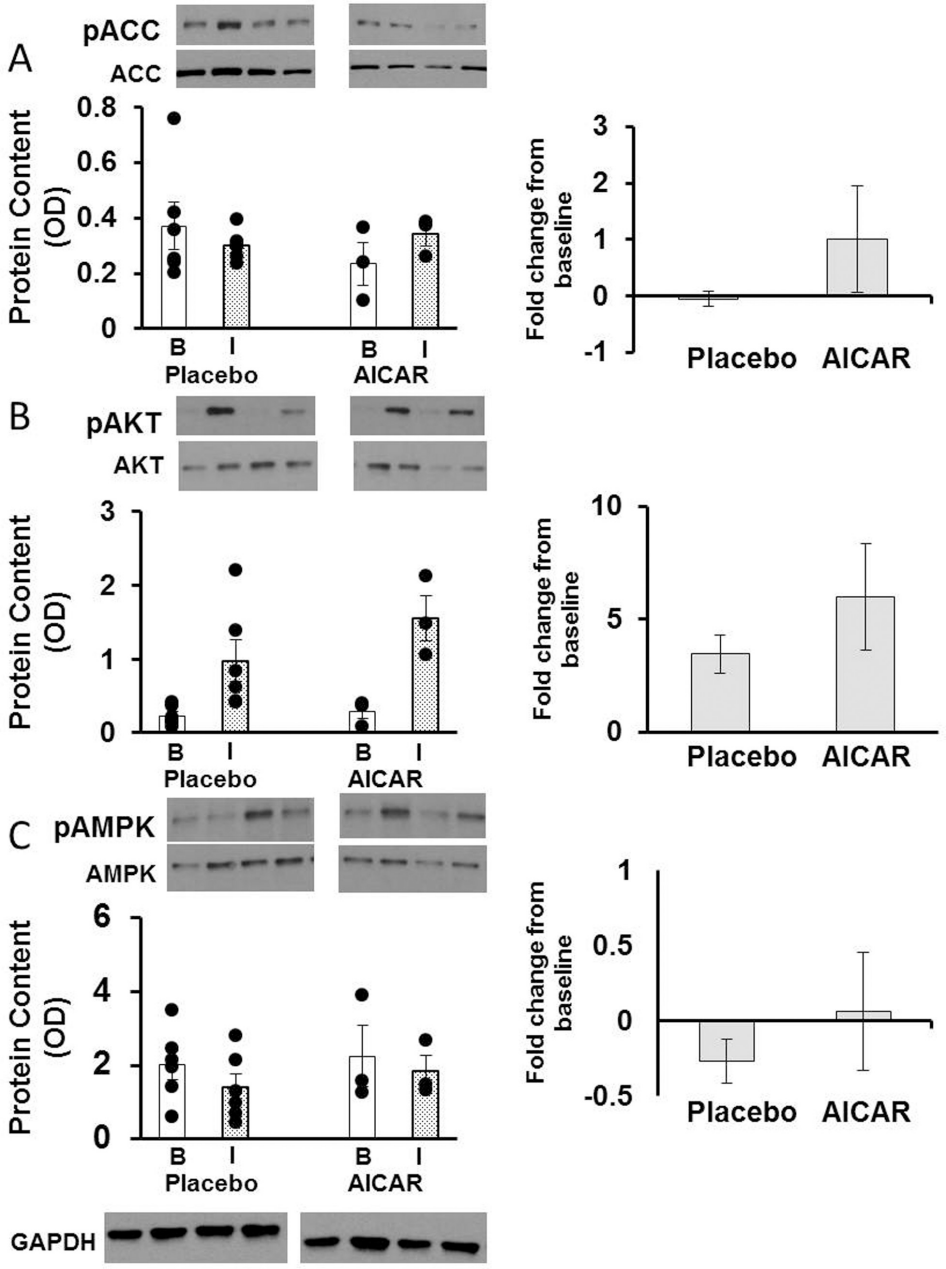
Skeletal muscle content of insulin signaling proteins in premature baboons on DOL 14. Skeletal muscle protein content of phosphorylated protein at baseline (clamp time +0 minutes, B; white column) and under insulin stimulation (clamp time +120 minutes, I; dotted column) and fold change from baseline to insulin-stimulated levels are shown for (A) ACC, (B) AKT, and (C) AMPK. Representative western blots are shown above their respective graphs. A representative loading control (GAPDH) blot is also shown. Error bars represent ± standard error of the mean.

mRNA content of *ACACA* in the skeletal muscle of preterm baboons was higher after AICAR infusion at baseline (p = 0.026, Fig 4A), but was similar between placebo and AICAR treated animals under insulin stimulation (p = 0.19, Fig 4A). The protein content of phosphorylated ACC was similar between groups (phosphorylated protein content normalized to total protein content); furthermore, the fold change under insulin-stimulated conditions from baseline was not significantly different between placebo and AICAR treated animals (p = 0.15, Fig 5A).

AICAR infusion significantly increased mRNA content of *AKT* in the skeletal muscle at baseline and under insulin-stimulated conditions (baseline: p = 0.04 and insulin-stimulated: p = 0.02, Fig 4B). The protein content of phosphorylated AKT was not significantly different between groups (baseline: p = 0.61 and insulin-stimulated: p = 0.26, Fig 5B); the fold increase from baseline was 3.5 in animals receiving placebo vs. 5.9 in animals receiving AICAR, but did not reach statistical significance (p = 0.27, Fig 5B).

The mRNA content of *PRKAA2* was not significantly different after 5 days of AICAR infusion at baseline (p = 0.12, Fig 4C), or under insulin stimulation (p = 0.06, Fig 4C). No difference in the protein content of phosphorylated AMPK was observed in placebo versus AICAR treated baboons at baseline (p = 0.77) or under insulin stimulation (p = 0.51, Fig 5C). The fold change was also similar between groups and exhibited high variability (Fig 5C).

mRNA content of *PPARGC1A* in the skeletal muscle of preterm baboons was similar between groups at baseline (p = 0.16), but was significantly higher in AICAR treated animals under insulin-stimulated conditions (p<0.01, Fig 4D).

**Fig 6:**
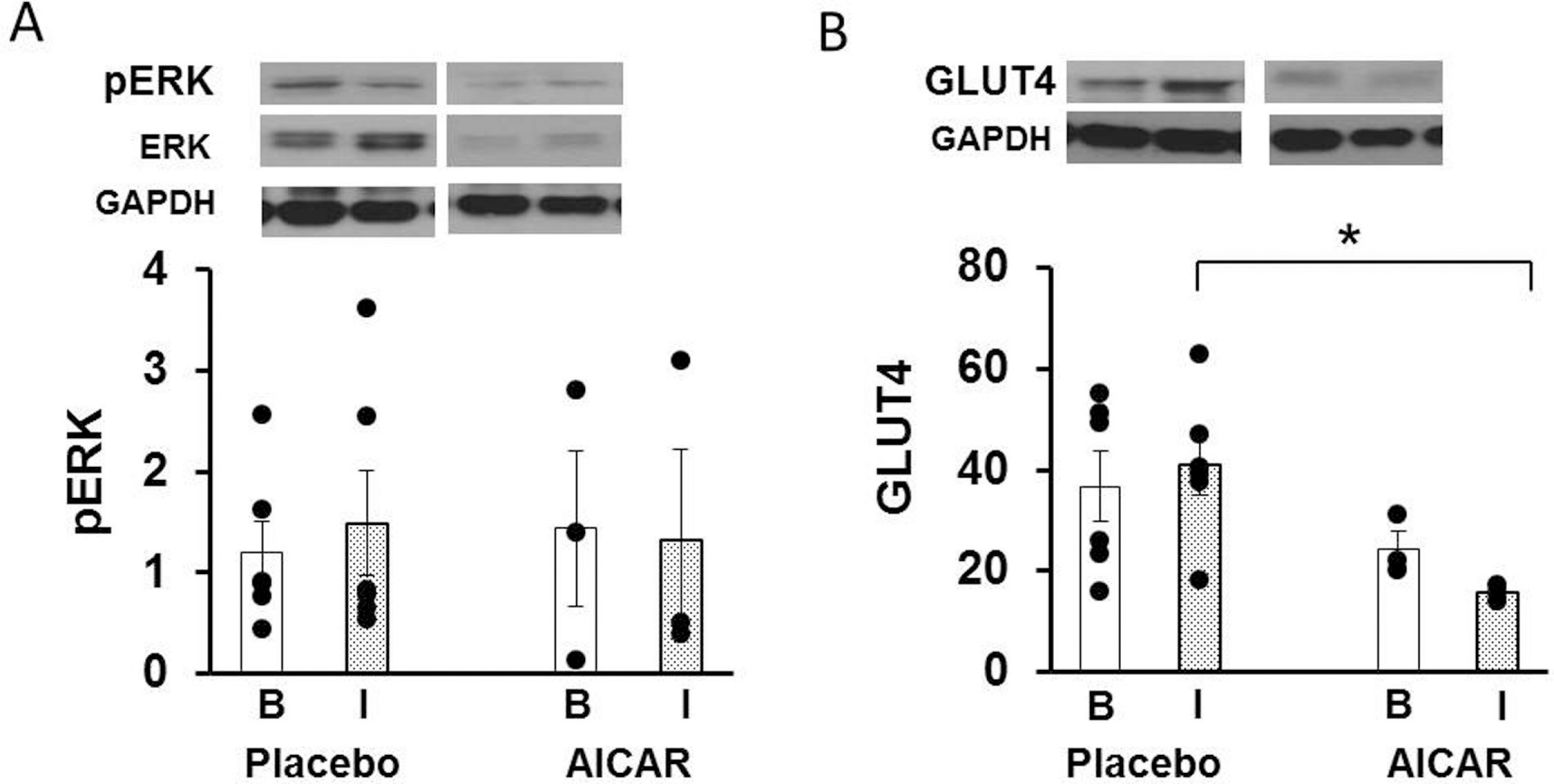
Protein content of insulin signaling molecules in skeletal muscle of premature baboons on DOL 14. Protein content of (A) phosphorylated ERK and (B) GLUT4 in the skeletal muscle of preterm baboons is shown at baseline insulin levels (clamp time +0 minutes, B; white bars), and under insulin stimulation (clamp time +120 minutes, I; dotted bars) for both treatment groups. Representative western blots are shown above their respective graphs. Representative loading control (GAPDH) western blots are also shown. Error bars represent ± standard error of the mean. *, P<0.05, placebo vs AICAR, independent samples T-Test.

Protein content of phosphorylated ERK in skeletal muscle was similar between animals receiving placebo vs animals receiving AICAR infusion at baseline (p = 0.73) and under insulin-stimulated conditions (p = 0.87, Fig 6A).

Skeletal muscle protein content of GLUT4 was significantly decreased after AICAR infusion under insulin-stimulated conditions (p = 0.023), whereas it was similar between groups at baseline (p = 0.28, Fig 6B).

#### Insulin signaling and gluconeogenic molecules in liver

**Fig 7:**
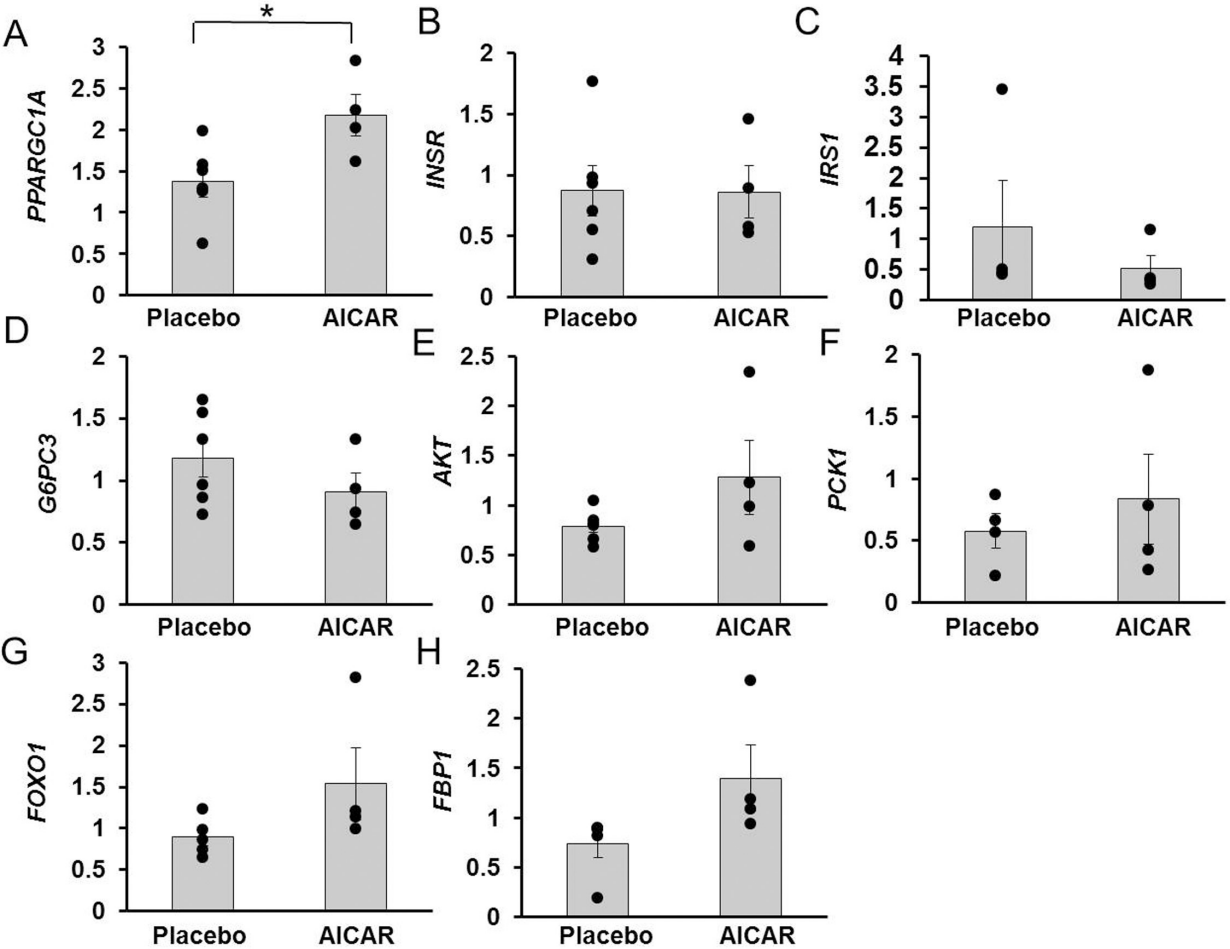
Liver relative mRNA content of insulin signaling and gluconeogenic molecules in premature baboons on DOL 14. Relative mRNA content of (A) *PPARGC1A*, (B) *INSR*, (C) *IRS1*, (D) *G6PC3*, (E) *AKT*, (F) *PCK1*, (G) *FOXO1*, and (H) *FBP1* in the liver of preterm baboons is shown for placebo and AICAR treated animals. Error bars represent ± standard error of the mean. *, p < .05, placebo vs AICAR, independent samples T-Test.

mRNA content of *PPARGC1A* was significantly increased in the liver after AICAR infusion in preterm baboons compared to placebo-treated animals (p = 0.03, Fig 7A).

**Fig 8:**
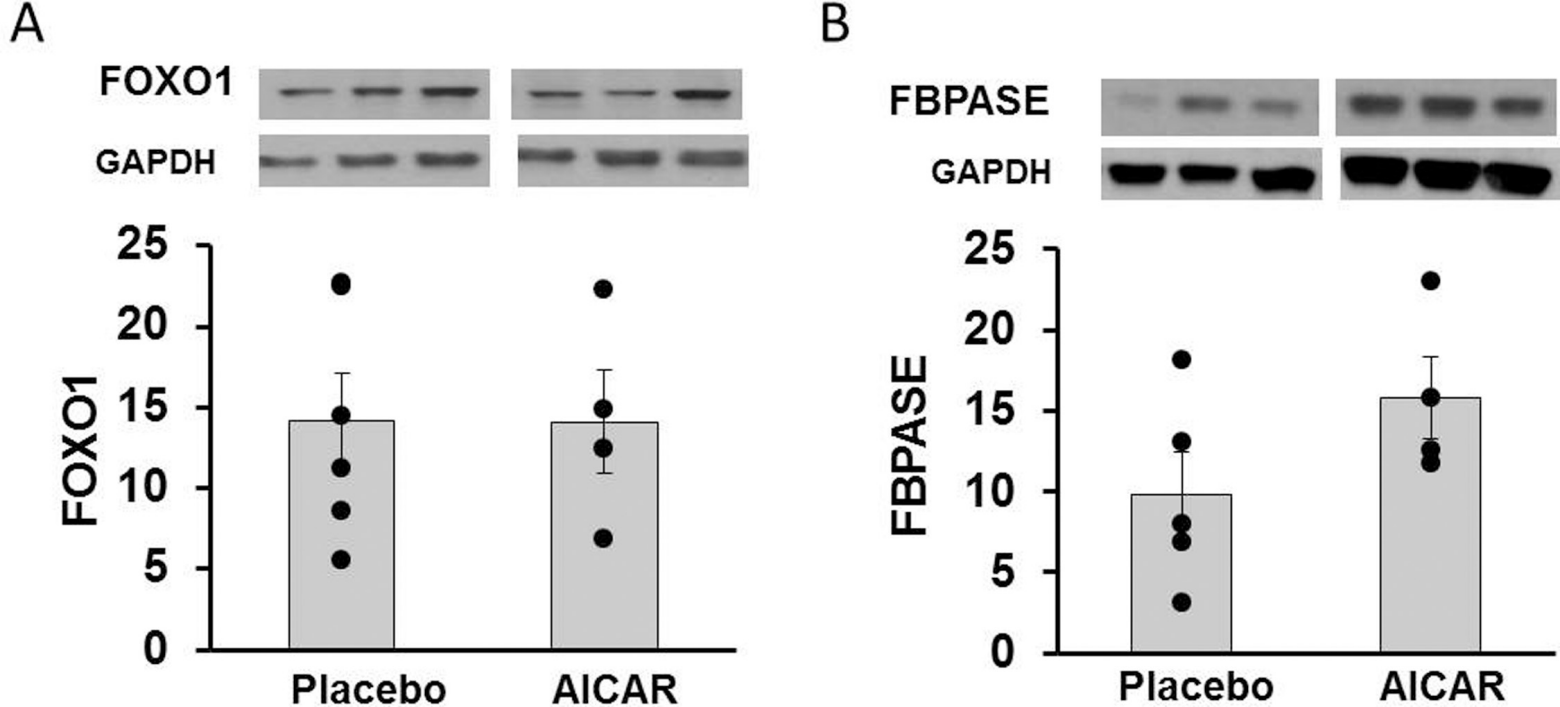
Protein content of insulin signaling molecules in the liver of premature baboons on DOL 14. Protein content of (A) FOXO1 and (B) FBPASE in the liver of preterm baboons is shown for both treatment groups. Representative western blots are shown above their respective graphs. Representative loading control (GAPDH) western blots are also shown. Error bars represent ± standard error of the mean.

Liver mRNA content of *INSR*, *IRS1*, *G6PC3*, *AKT*, *PCK1*, *FOXO1*, and *FBP1* was not significantly different in animals receiving AICAR versus placebo infusion (p = 0.97, p = 0.42, p = 0.27, p = 0.15, p = 0.53, p = 0.15, and p = 0.09, Fig 7B–7H, respectively). Finally, protein content of FOXO1 and FBPASE was not significantly different between AICAR and placebo-treated baboons (p = 0.99, p = 0.15, Fig 8A and 8B, respectively).

Because there was no significant effect after 5 days of AICAR infusion on insulin sensitivity in the first animals examined, the final two animals had the first insulin clamp soon after AICAR was initiated. This was done in order to examine the effects of AICAR stimulation right after the initial bolus. This was then followed by 5 days of AICAR stimulation and the second insulin clamp on DOL 14, exactly the same as the previous animals.

Insulin sensitivity (*M*-value) did not improve after 90 minutes of AICAR infusion (*M*-value 12.8±1.6 vs 11.6±2.5 mg/(kg·min), placebo vs AICAR, respectively, DOL 5). However, there were some effects observed after the initial bolus of AICAR infusion. The skeletal muscle protein content of phosphorylated ACC, AMPK, and AKT seemed to increase after 90 minutes of AICAR bolus of 0.15 mg/g followed by a continuous infusion of 0.5 mg·g^-1^·day^-1^ when compared to placebo under insulin-stimulated conditions. However, this was not statistically significant (p = 0.34, p = 0.15, p = 0.42, respectively, S1 Fig) and this effect was not sustained after 5 days of AICAR infusion (Fig 5).

## Discussion

In this study, we investigated the effects of 5 days of AICAR infusion on insulin sensitivity in premature euglycemic baboons utilizing the euglycemic hyperinsulinemic clamp procedure. We found no effect in the peripheral tissue sensitivity to insulin after 5 days of AICAR infusion. We also aimed to understand the mechanisms of AICAR effects on insulin sensitivity; therefore, we measured key insulin signaling proteins in skeletal muscle. AICAR infusion did not significantly increase phosphorylation of AMPK, nor did it increase mRNA expression of *PRKAA2*. These results are in contrast to other animal data, which have shown activation when directly injected into the rat muscle and after chronic intravenous infusion [32,33]. The lack of effect might be due to the inability to achieve serum AICAR concentrations in our premature baboons similar to those previously reported in adult systems. Additionally, one animal developed renal failure during treatment with higher dosing of AICAR. Following these issues, the study was terminated early due to ethical and safety concerns, and remains underpowered for hard conclusions. However, we believe the study still allows meaningful observations to be made concerning insulin signaling and insulin resistance in premature baboons. Our results are consistent with human data where AICAR failed to increase AMPK phosphorylation in diabetic adults [25] or in healthy men [26] whereas it increased after exercise, which is consistent with the idea that ZMP allosterically activates AMPK [22]. Finally, reduced response of AMPK to AICAR stimulation has been shown in the skeletal muscle of elderly rats, contributing to insulin resistance and other metabolic disturbances [34]. It remains to be determined, if similar to elderly rats, AMPK protein content and/or responsiveness in premature baboons is also low when compared to more mature animals.

AMPK phosphorylates ACC, leading to a decrease in its activity, and a subsequent increase in fatty acid oxidation [14]. Consistent with our findings of no improvement in AMPK phosphorylation with AICAR infusion, we found no improvements in phosphorylation of ACC following AICAR infusion. AICAR infusion did increase mRNA expression of *ACACA*, however these changes did not translate into improved protein phosphorylation. Furthermore, we found no significant differences in serum FFA levels in AICAR versus placebo treated baboons (mean ± SD: 0.10±0.04 vs 0.21±0.16 meq/L, respectively, p = 0.3).

AICAR acts to lower serum glucose by increasing glucose uptake and inhibiting gluconeogenesis [24,35]. However, in this study we found no differences in serum glucose levels or glucose infusion rates to maintain euglycemia, suggesting AICAR treatment had no effect on glucose uptake or gluconeogenesis in premature euglycemic baboons. This result coincides with the findings of Bosselaar et al, wherein AICAR infusion did not increase skeletal muscle glucose uptake in healthy adults [36]. Contrary to our hypothesis, protein content of GLUT4 was reduced in AICAR treated animals. In the insulin signaling cascade, activation of AMPK by AMP, or ZMP, is expected to induce translocation of GLUT4 to the surface membrane, which then increases glucose uptake in striated muscle and adipose tissue. Therefore, the lack of GLUT4 increase is likely due to the poor response of AMPK activation after AICAR infusion.

PCG1α is another key metabolic regulatory protein, though its contribution to insulin sensitivity/resistance is less well understood [13]. Increased expression of *PPARGC1A* in skeletal muscle has been demonstrated to increase *SLC2A4* expression, and consequently improve insulin sensitivity *in vitro* [37]. *In vivo*, massive over-expression of *PPARGC1A* was associated with insulin resistance; however, a modest increase in expression, as seen with exercise, improves insulin sensitivity [38]. Furthermore, in transgenic mice, over-expressing *PPARGC1A* improved insulin sensitivity by elevating insulin-stimulated AKT phosphorylation [39]. In the present study, we detected a modest, but significant increase in *PPARGC1A* mRNA expression in the skeletal muscle after AICAR infusion under insulin-stimulated conditions. Although we did observe a significant increase in mRNA expression of *AKT* at baseline and under insulin-stimulated conditions in AICAR treated animals, we did not observe a corresponding increase in either AKT phosphorylation or insulin sensitivity.

Because AICAR also affects glucose homeostasis pathways in the liver, we examined protein and mRNA content of key molecules in the gluconeogenesis and insulin signaling pathways in the liver. Although not part of the original aims of the study, this data helps to illuminate the lack of improvement in insulin sensitivity with AICAR treatment. In the liver, *PPARGC1A* mRNA expression was significantly increased by AICAR treatment. *PPARGC1A* overexpression in liver has been associated with hepatic insulin resistance, manifested by higher glucose production and impaired suppression of gluconeogenesis in response to insulin in transgenic mice [39]. The degree to which these seemingly conflicting effects of *PPARGC1A* contributed to the inability of AICAR infusion to improve insulin sensitivity cannot be determined at this time as this was not the primary aim of this study. FBPASE has been postulated to be a key molecule by which AICAR inhibits gluconeogenesis [22]. Although there seemed to be a trend towards increased mRNA expression of *FBP1* and protein content of FBPASE in the AICAR treatment group, this effect was not significant (p = 0.09 and p = 0.15, respectively). We also found no differences in hepatic mRNA expression of other gluconeogenic enzymes (Fig 7), and no differences in protein content of FOXO1 between placebo and AICAR treated animals (Fig 8).

In patients with type 2 diabetes, intravenous AICAR administration decreased systemic glucose and FFA levels [25]; contrary to these results, we found no differences in systemic glucose levels/glucose infusion rates nor did we find differences in FFA. On the other hand, the preterm baboons remained euglycemic by providing limited glucose infusion and therefore these results cannot be extrapolated to AICAR effects under hyperglycemic conditions. Finally, glycogen content in the muscle and liver was similar between placebo and AICAR infused animals, suggesting that the lack of AICAR effect is not mediated by alterations in glycogen content. Similarly, AICAR was found to not affect glycogen concentrations in healthy adults [26].

As previously stated, a limitation in this study was achieving serum AICAR concentrations comparable to those found to improve insulin sensitivity and glucose control in other systems [26]. Despite careful calculations mimicking prior animal and human studies, close monitoring, and adjustment of AICAR dosing, we were unable to achieve concentrations similar to those achieved in adult humans (0.23 mmol/L). This is likely due to metabolic differences related to prematurity and differing pharmacokinetics of AICAR in preterm infants compared to adults. To our knowledge, no study examining AICAR metabolism in preterm humans/ animals exists. After initially undershooting the target, adjusting to a higher dose resulted in acute renal failure consisting of symptomatic hyperkalemia, oliguria, and uremia. This is not the first report of higher doses of AICAR being associated with adverse effects. Merrill et al also reported liver and metabolic complications in rats dosed at 1 mg/g body weight [33]. Further adjustments of AICAR dosing close to the not-tolerated dose still did not achieve concentrations similar to adult humans, but did allow continuation of infusion without side effects. We recognize that the small sample size used for this study represents a major limitation. Furthermore, the need for continued adjustments to AICAR dosing in the treatment group and the need to include shared controls (that received no infusion) in the placebo group increases the sample heterogeneity. However, given the severe adverse effects encountered, the number of non-human primates killed for the experiments needs to remain limited. Additionally, although every effort was made to reduce the number of confounding variables, the requirement for mechanical ventilation, and the possibility of anoxia affecting glucose metabolism in these animals cannot be fully accounted for. Finally, although the animals were maintained with appropriate sedation and analgesia, a final confounder that might have affected the results of this study is stress and unforeseen pain. However, AICAR infusion did not alter lactate levels or counter-regulatory hormones, as they remained similar between treatment groups; further, counter-regulatory hormones remained stable under basal and insulin-stimulated conditions and therefore we conclude that stress was not likely a significant contributor to the lack of differences between groups.

In conclusion, this study provides evidence that treatment with AICAR does not improve insulin sensitivity in preterm, euglycemic, insulin-resistant baboons. At high doses, AICAR infusion may be associated with adverse effects. AICAR did not improve the phosphorylation of key insulin signaling proteins such as AKT, ACC, and AMPK or protein content of GLUT4. To our knowledge, this is the first time AICAR has been studied as an intravenous infusion in a premature non-human primate.

## Supporting information

S1 Images Western blot membranes used for analysis. Raw images of western blot membranes used in analysis are shown. Bands used for figures are also indicated.
(PDF)

S1 Fig Effects of 90 Minutes of AICAR stimulation in premature baboons. Western blot membranes demonstrating the effects of 90 minutes of AICAR infusion on skeletal muscle protein content of phosphorylated ACC, AMPK, and AKT are shown. B: baseline insulin levels (or clamp time +0 minutes), I: insulin-stimulated (or clamp time +120 minutes). A representative GAPDH loading control is also shown.
(TIF)
