# Supplementary material for: Effects of intravenous AICAR (5-aminoimidazole-4-carboximide riboside) administration on insulin signaling and resistance in premature baboons, Papio sp
Source: PLoS One. 2018 Dec 12;13(12):e0208757. doi: 10.1371/journal.pone.0208757 (PMC6291136; doi:10.1371/journal.pone.0208757)
Supplement: S1 Images — Raw images of western blot membranes used in analysis are shown. Bands used for figures are also indicated. (PDF) [file pone.0208757.s001.pdf]

AICAR MUSCLE

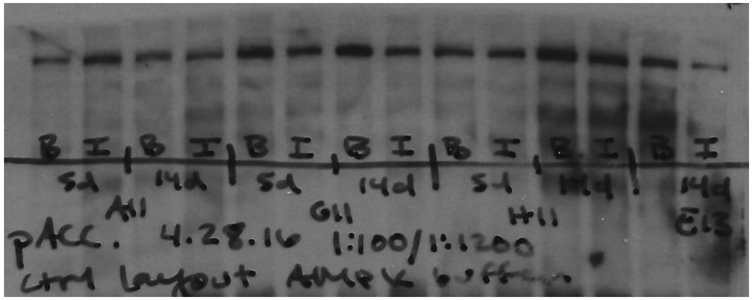

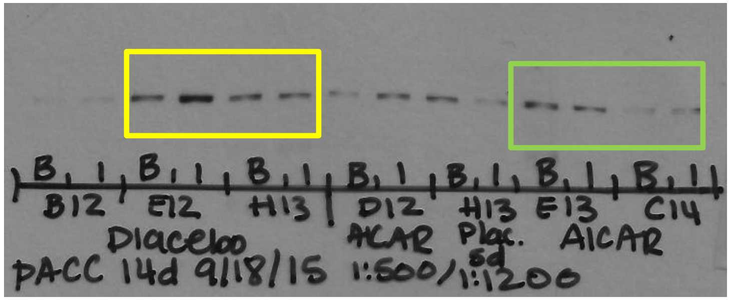

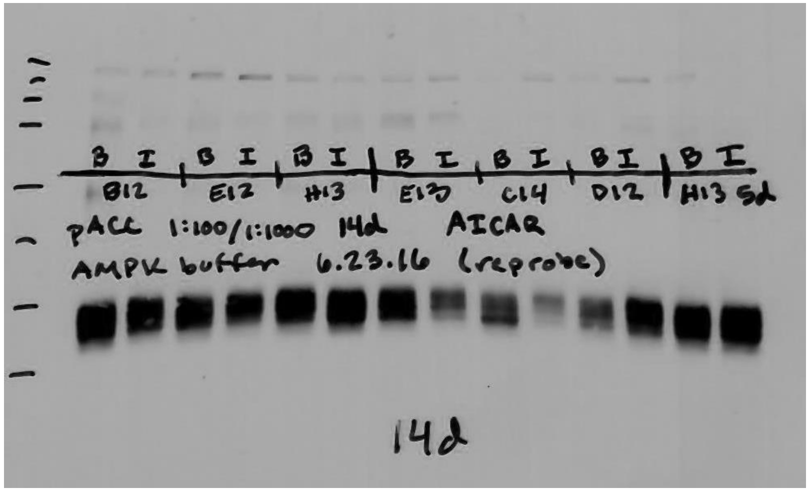

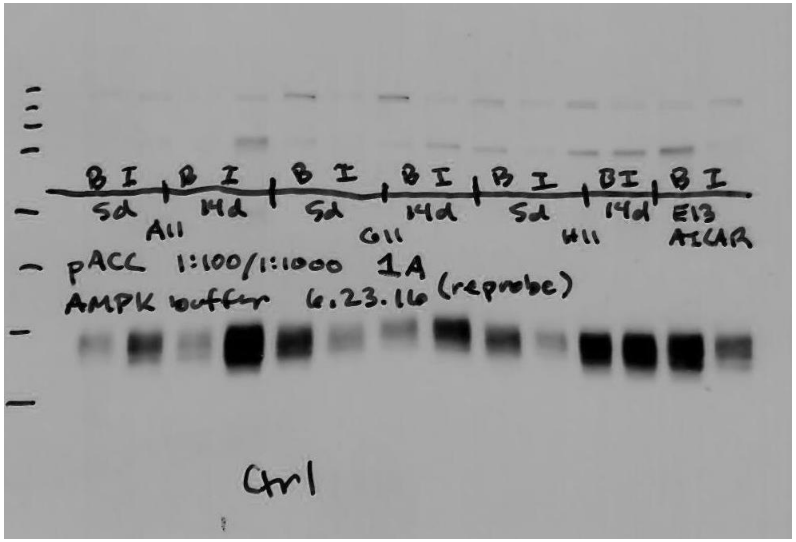

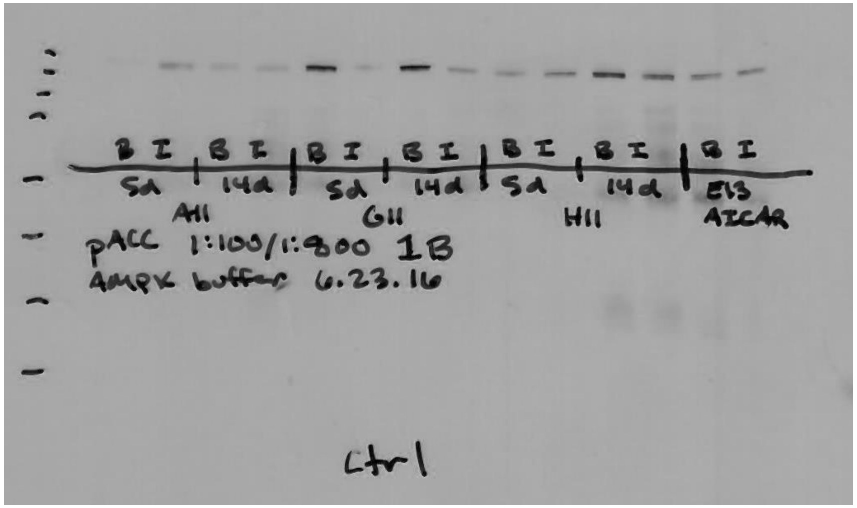

pACACA, Figure 5A

Placebo
AICAR

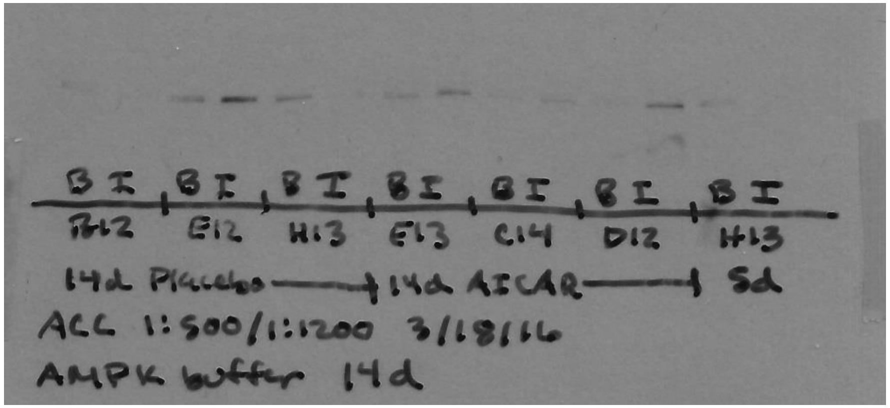

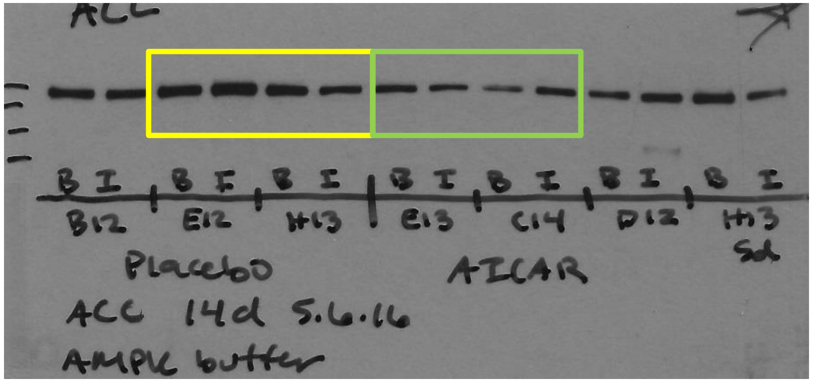

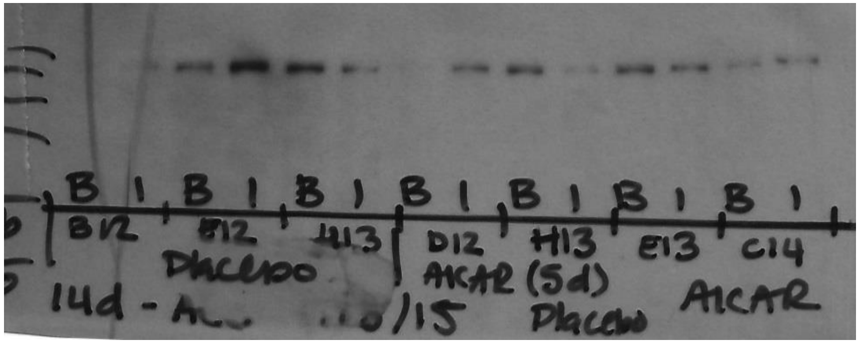

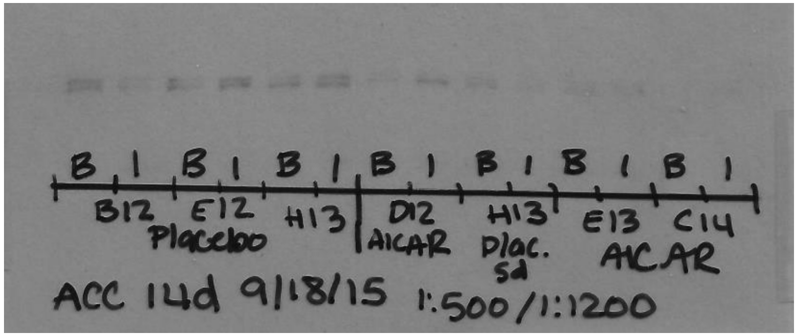

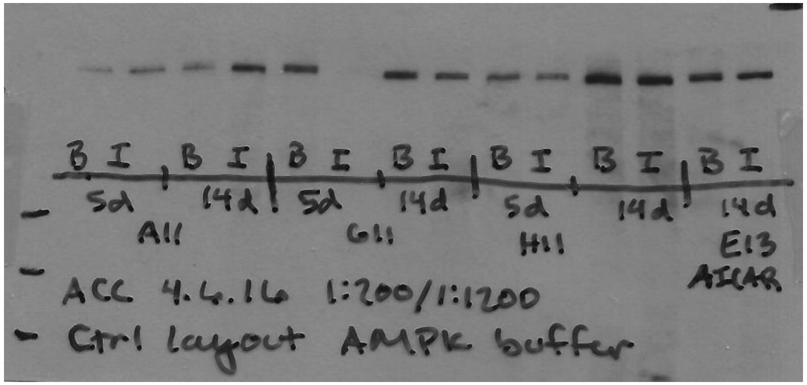

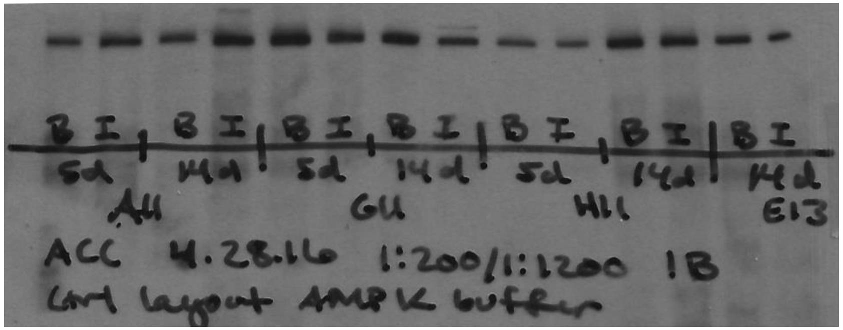

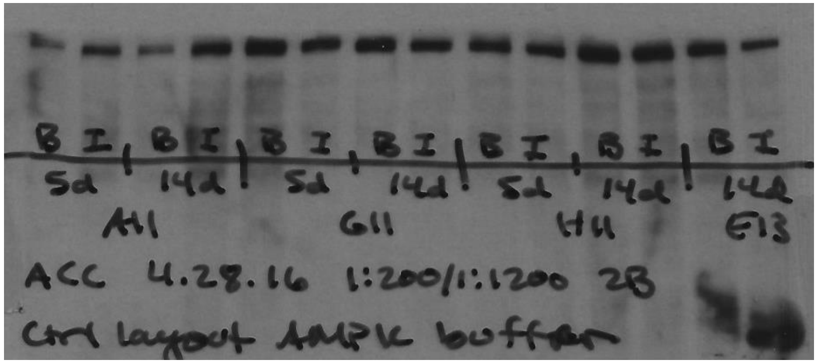

ACACA, Figure 5A

Placebo
AICAR

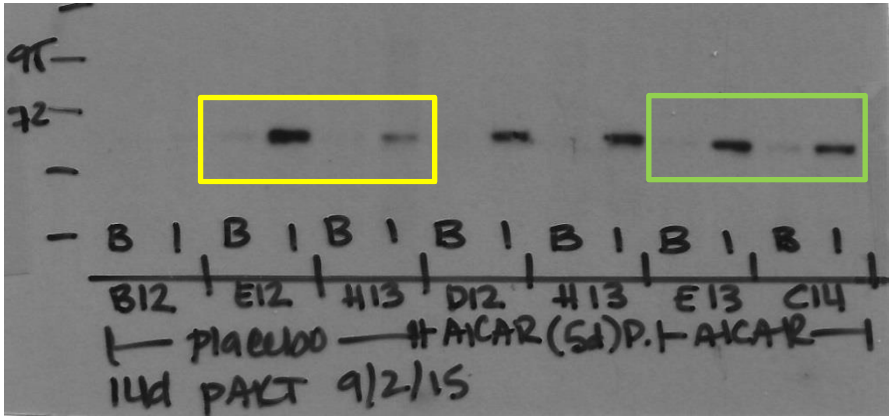

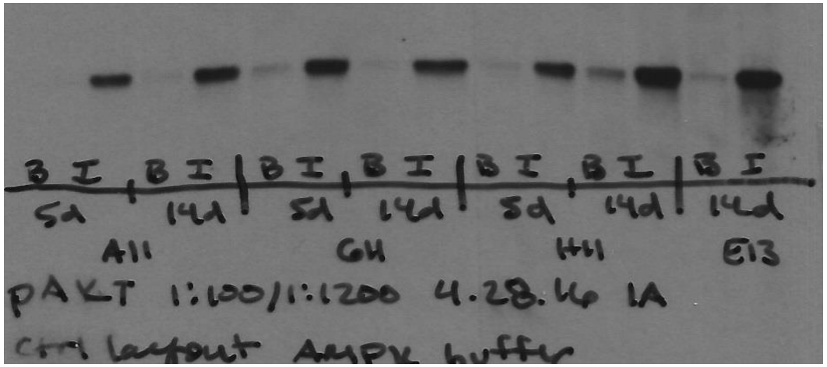

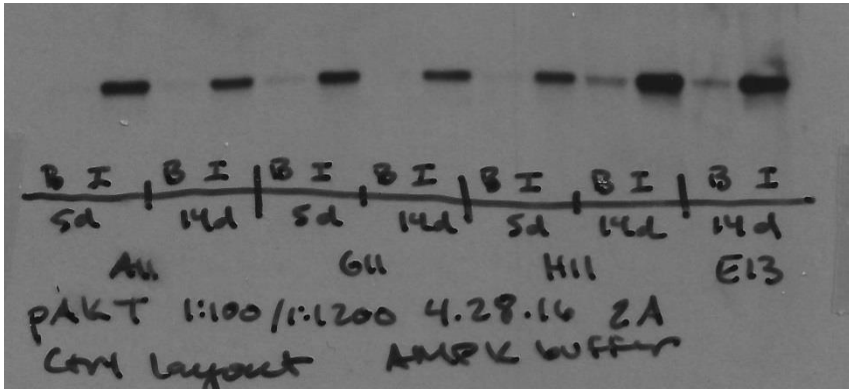

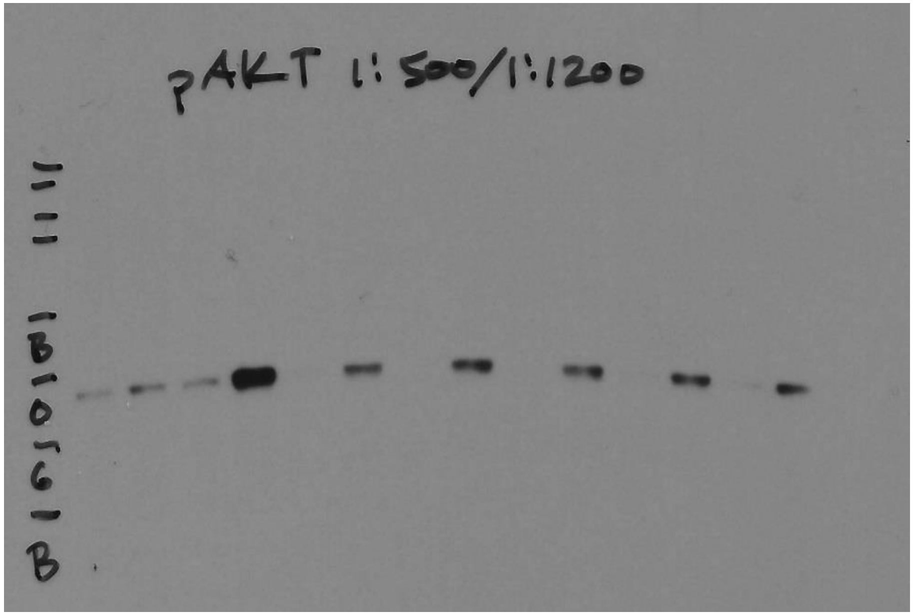

pAKT, Figure 5B

Placebo
AICAR

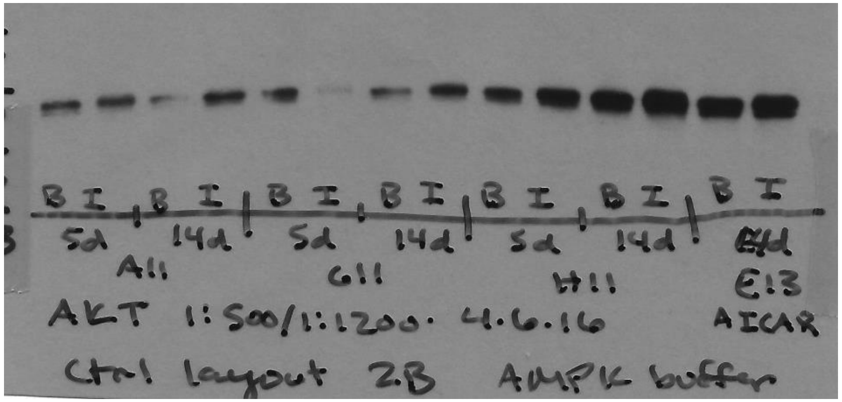

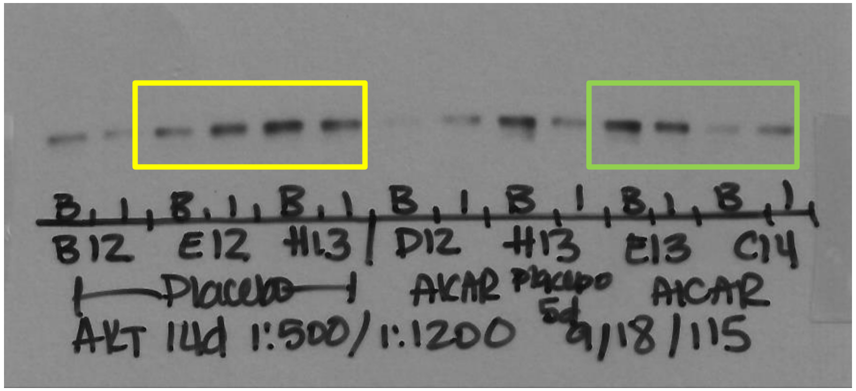

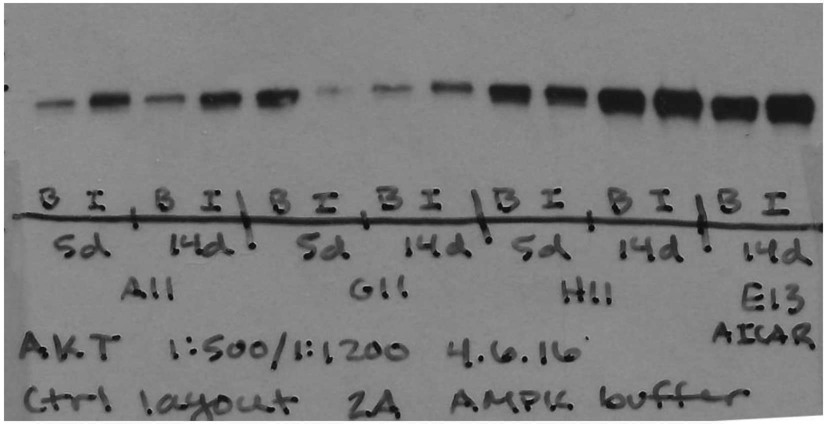

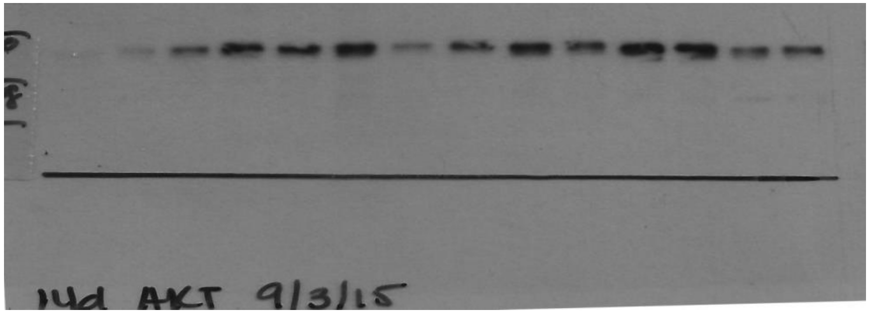

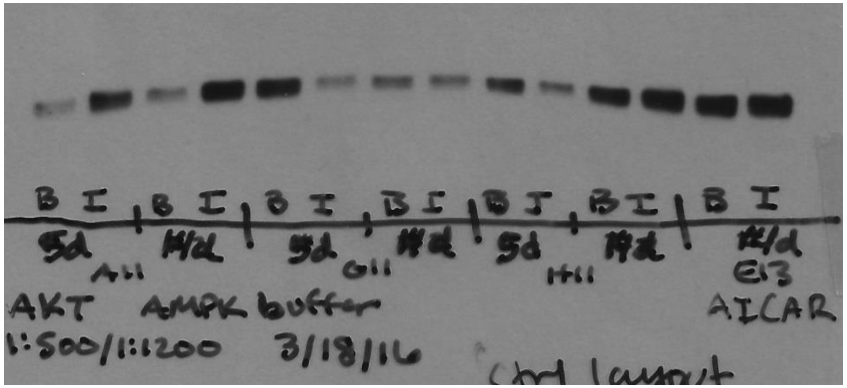

AKT, Figure 5B

Placebo
AICAR

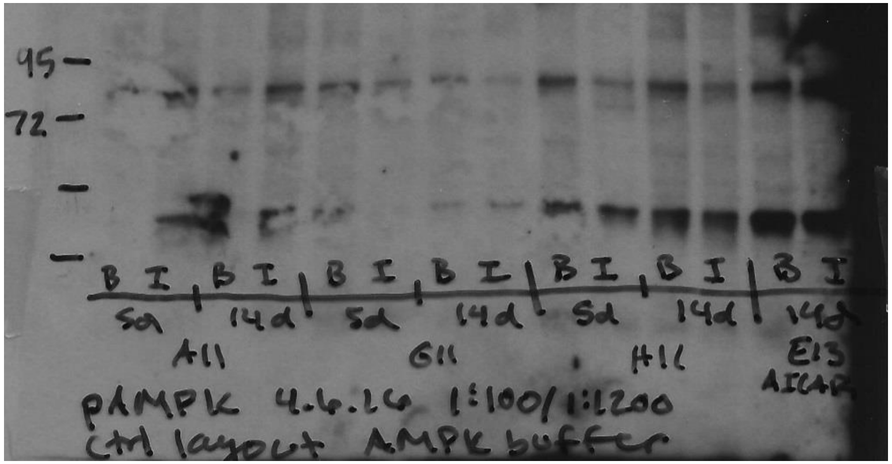

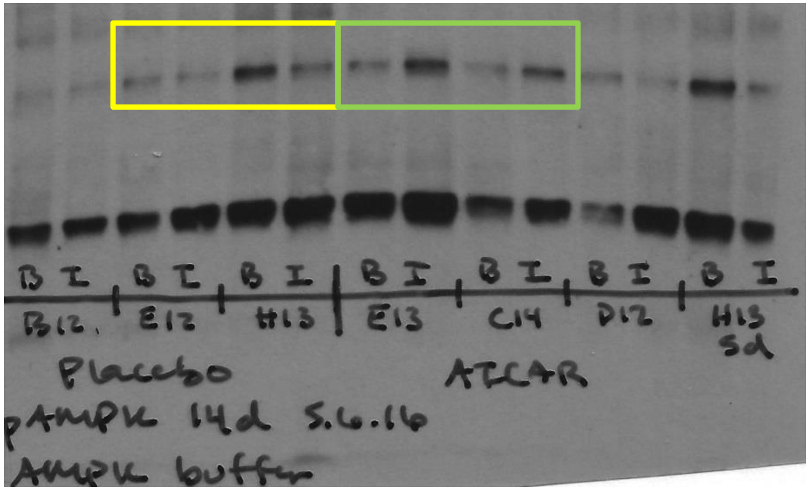

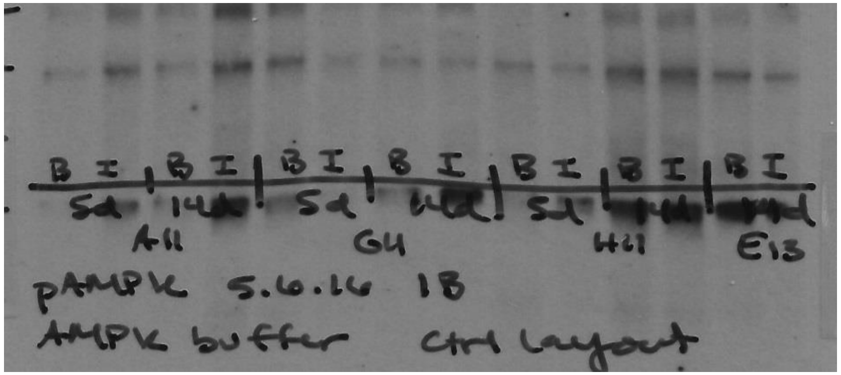

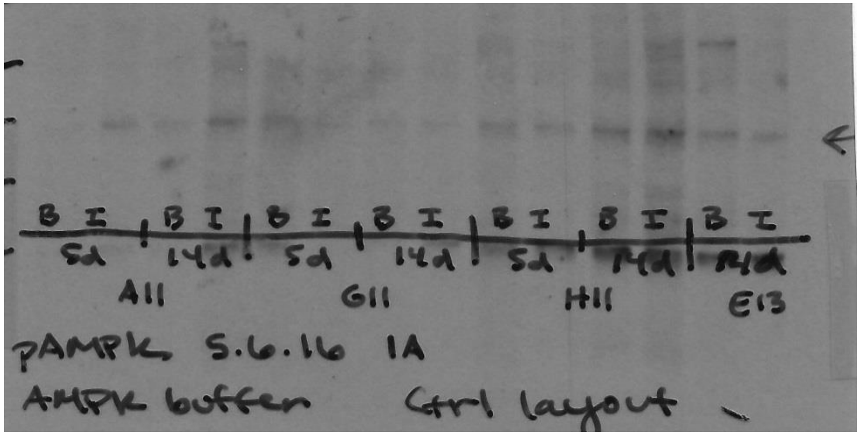

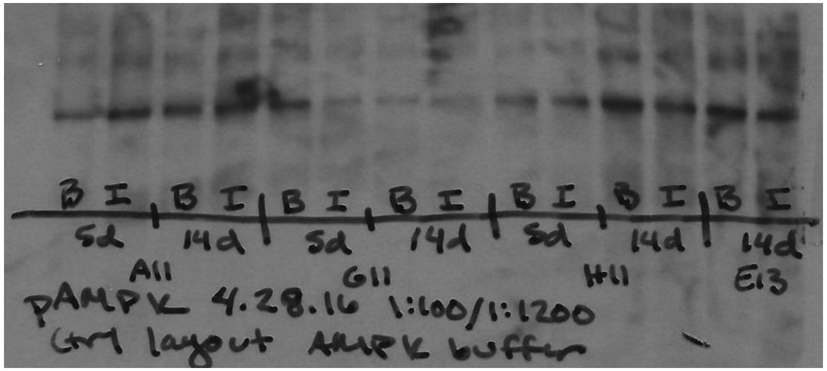

pPRKAA, Figure 5C

Placebo
AICAR

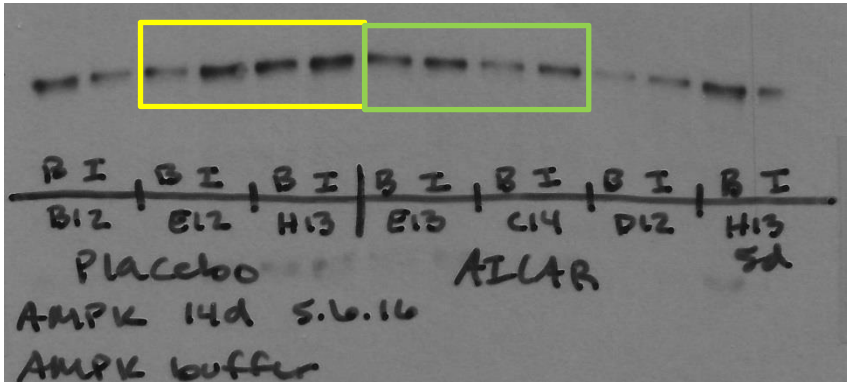

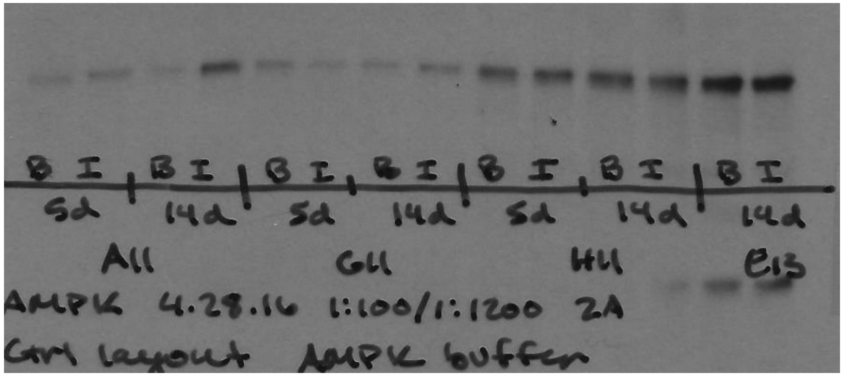

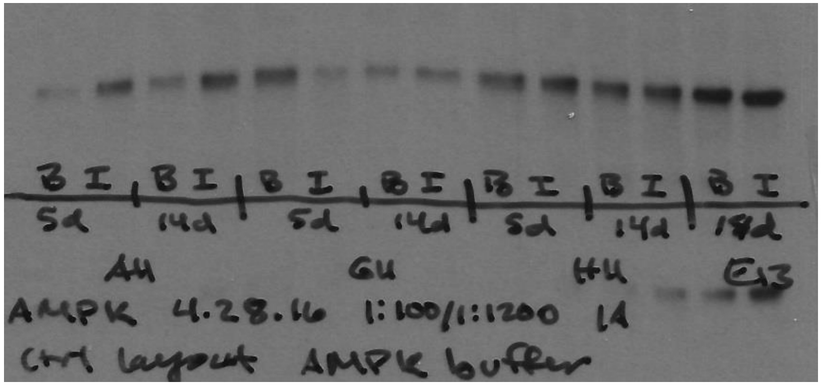

PRKAA, Figure 5C

Placebo
AICAR

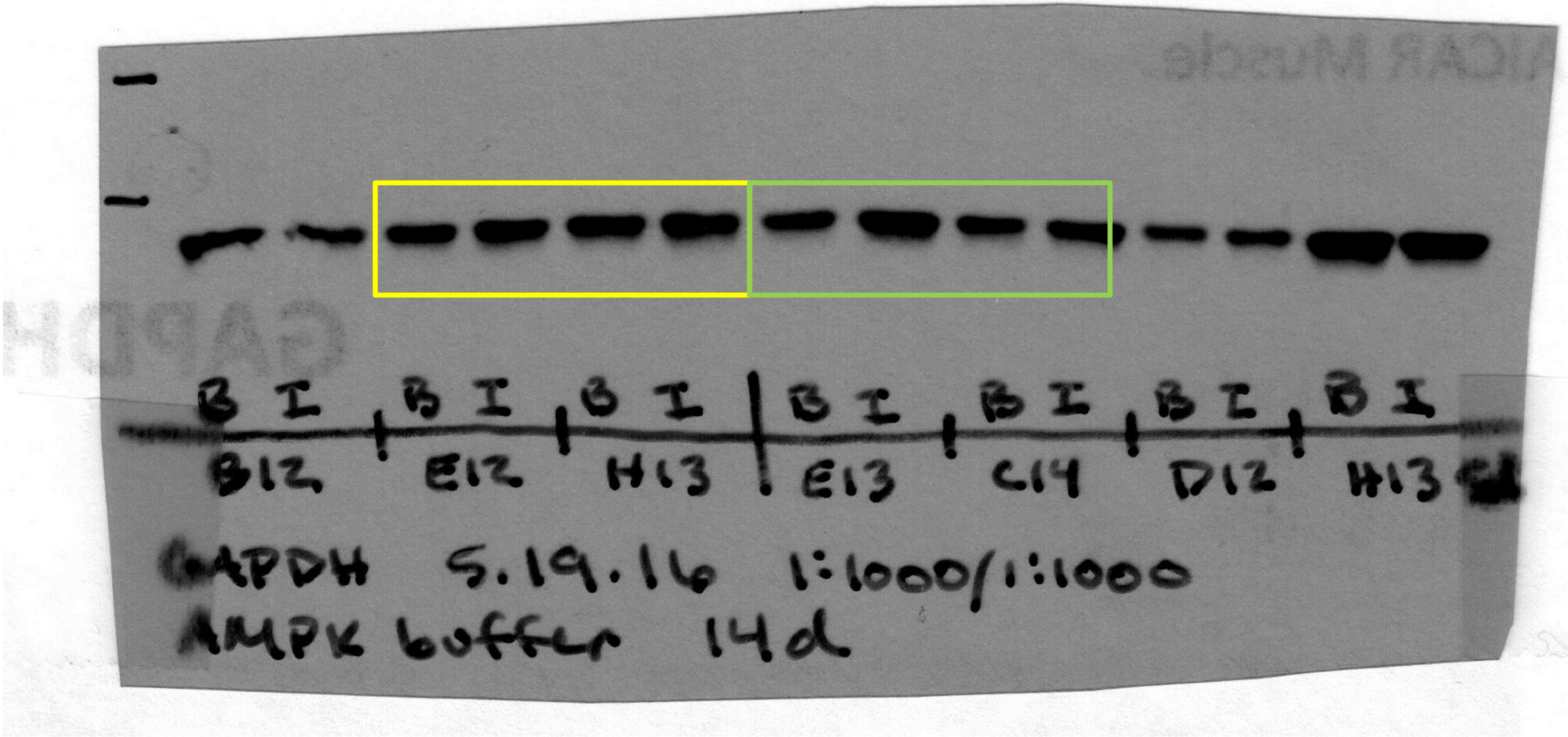

GAPDH, Figure 5

Placebo
AICAR

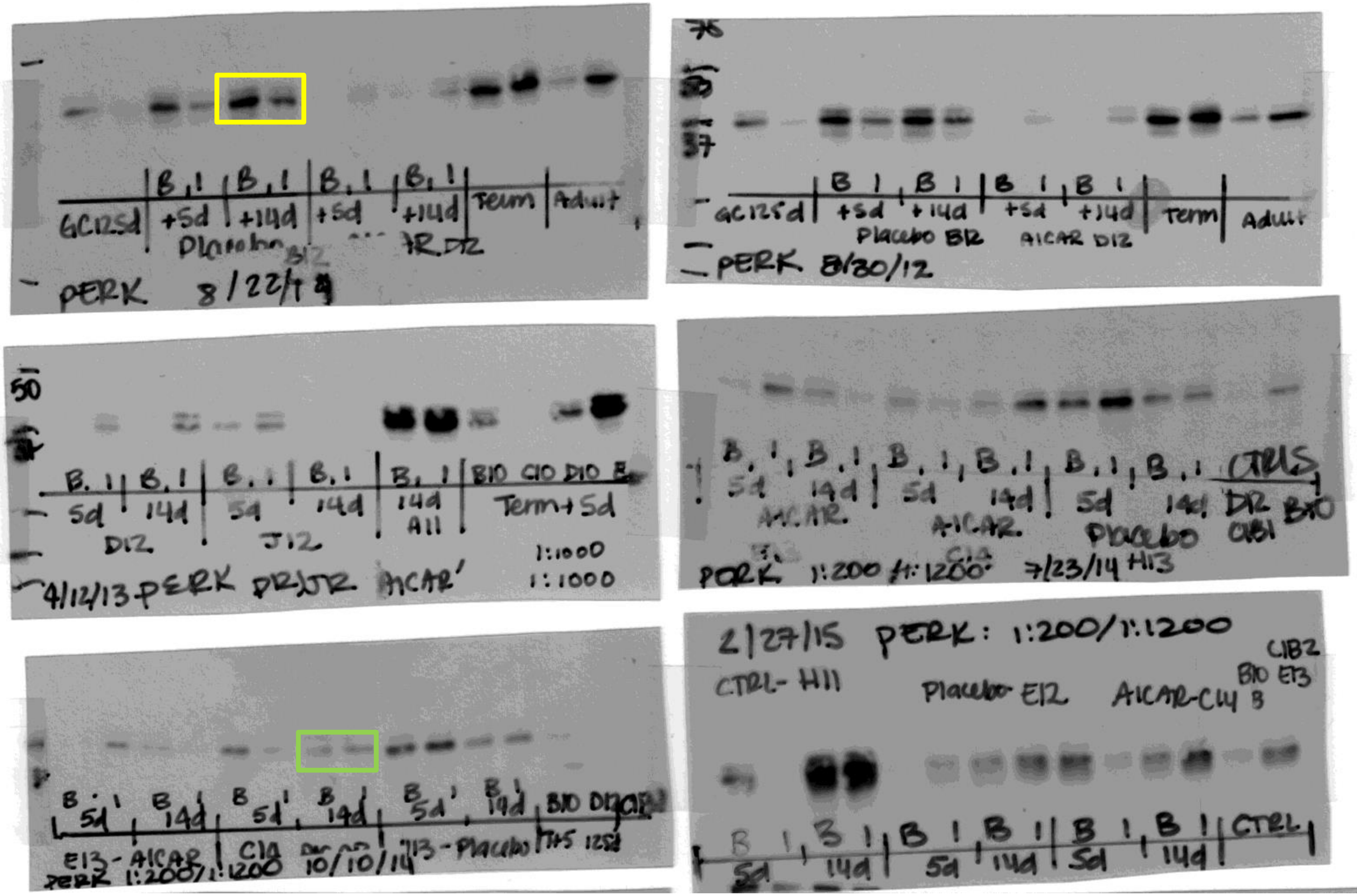

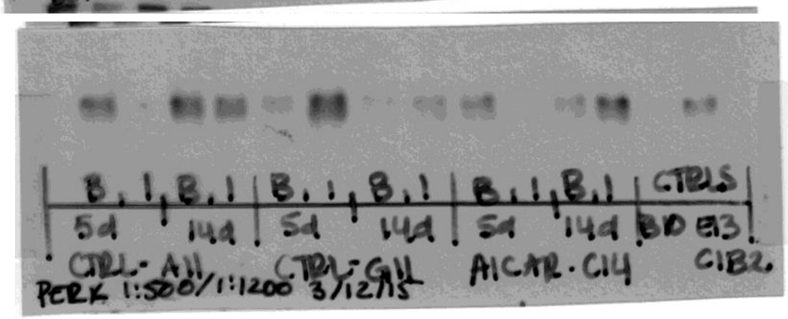

pMAPK1, Figure 6A

Placebo
AICAR

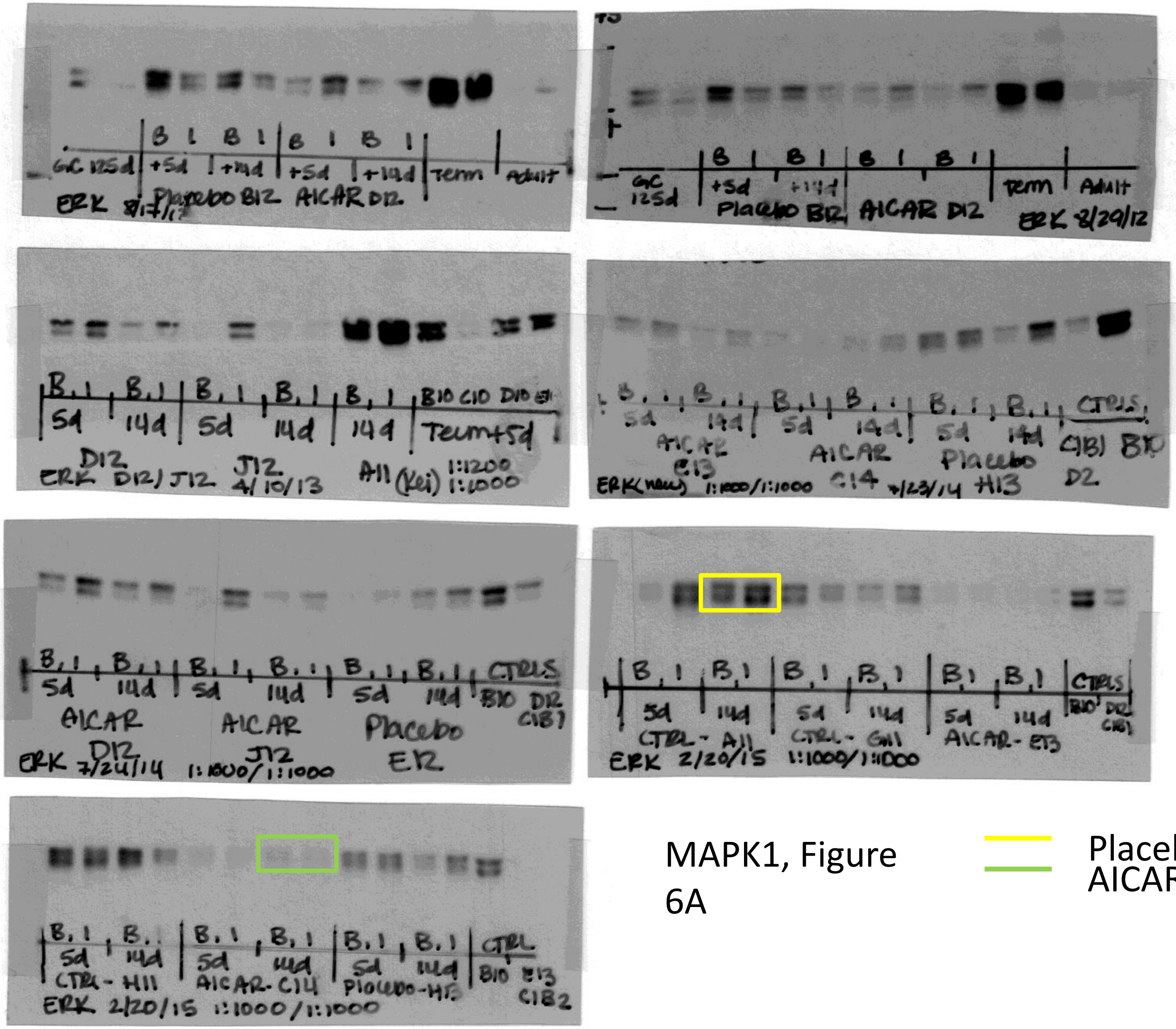

MAPK1, Figure 6A

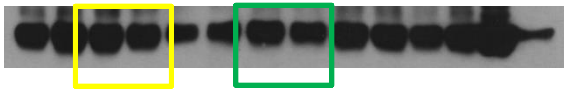

GAPDH MAPK1, Figure 6A

Placebo
AICAR

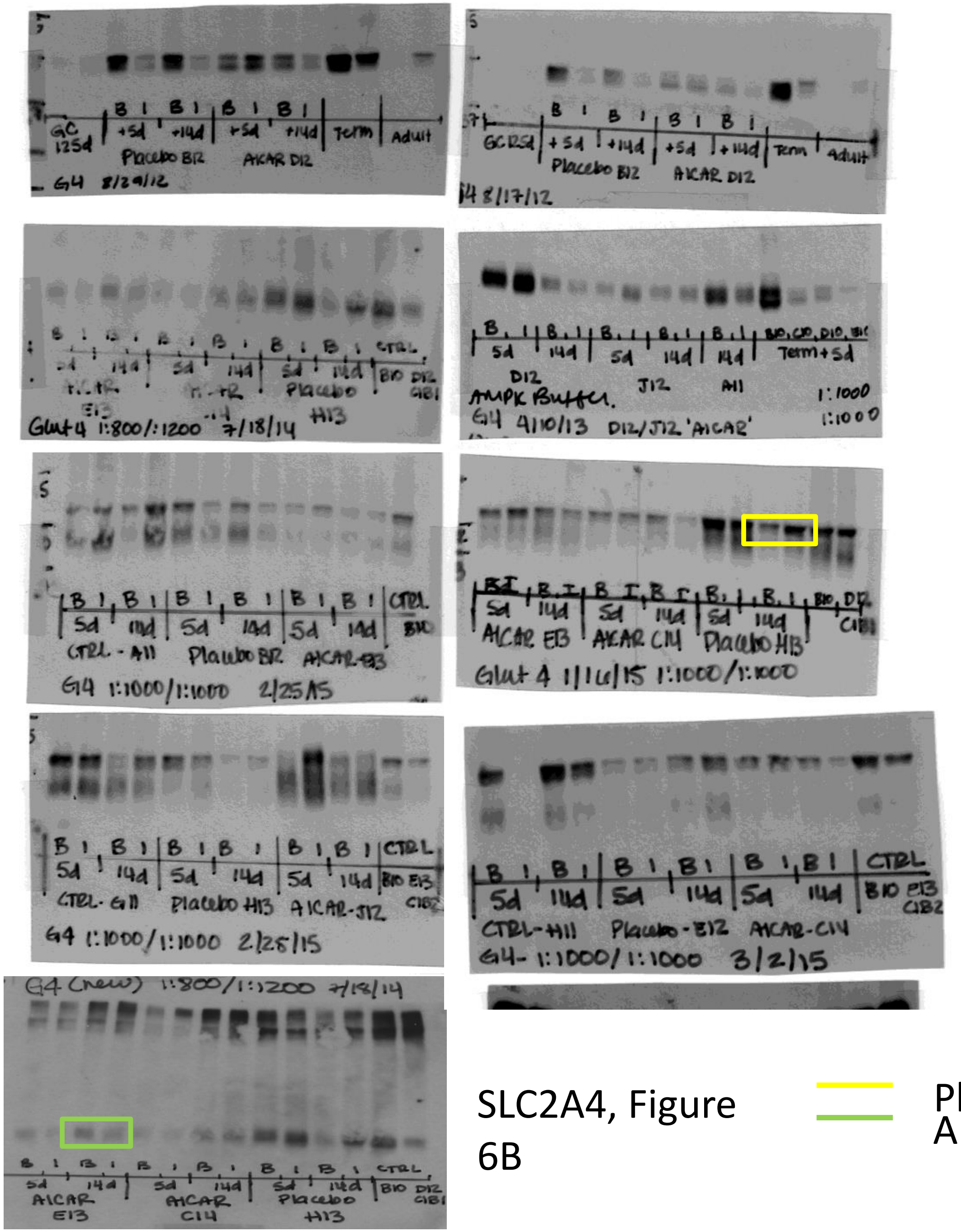

SLC2A4, Figure 6B

Placebo
AICAR

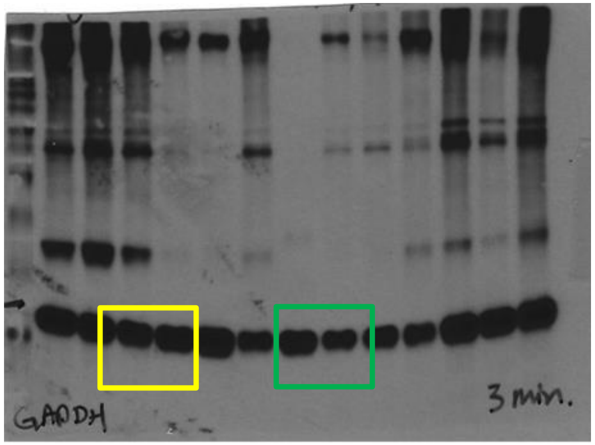

GAPDH, SLC2A4, Figure 6B

Placebo
AICAR

AICAR LIVER

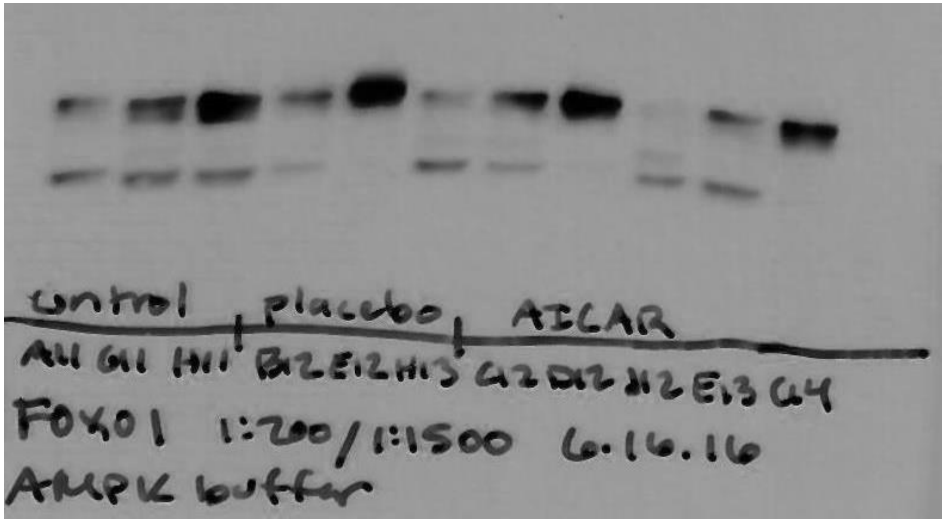

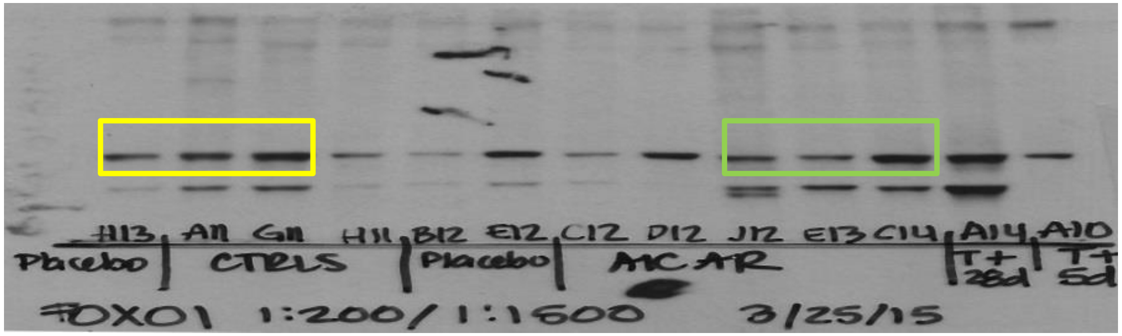

FOX01, Figure 6C

Placebo
AICAR

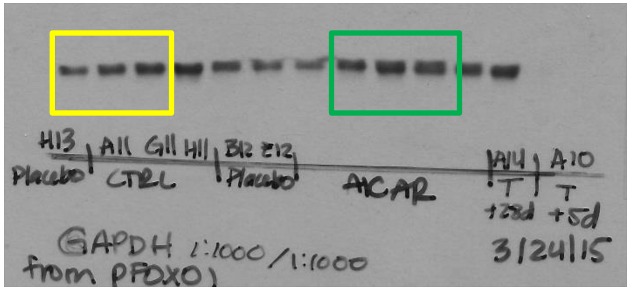

FOX01 GAPDH Figure 6C

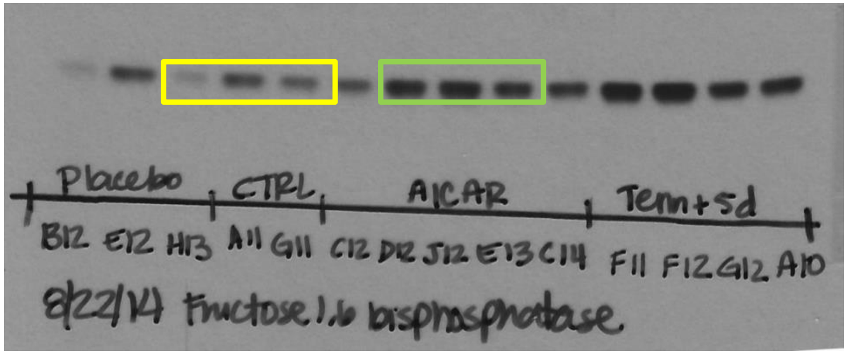

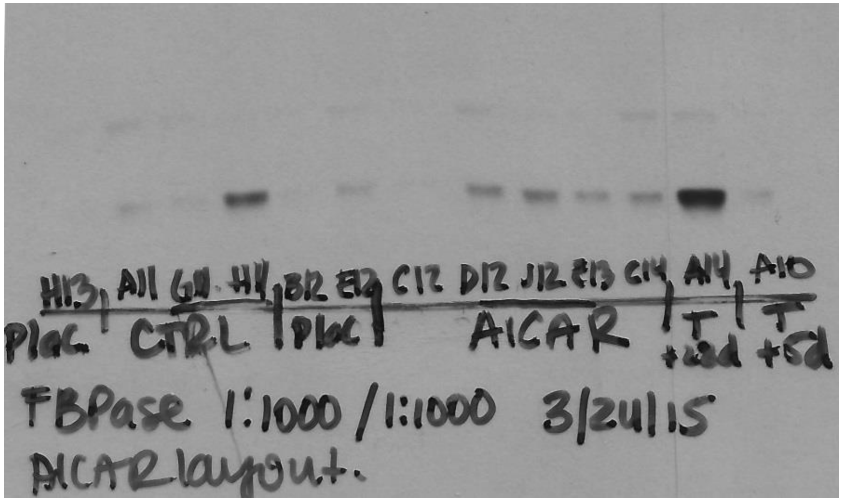

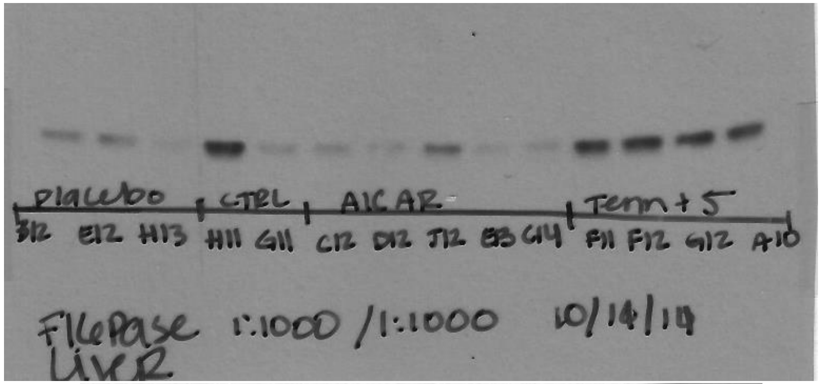

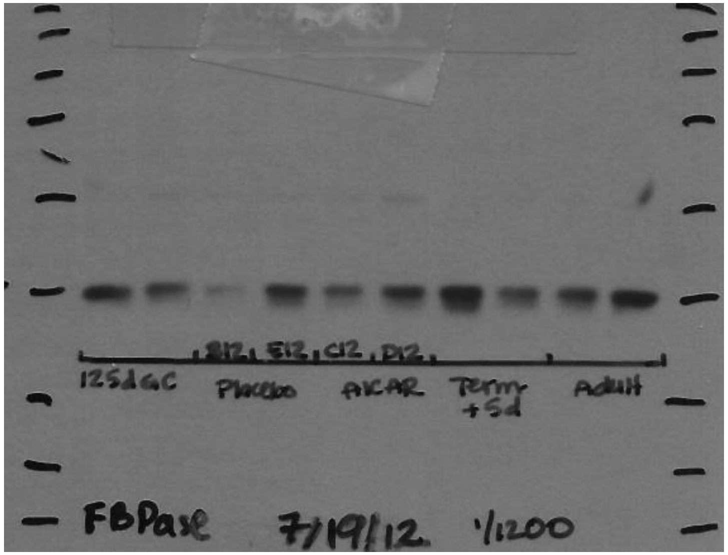

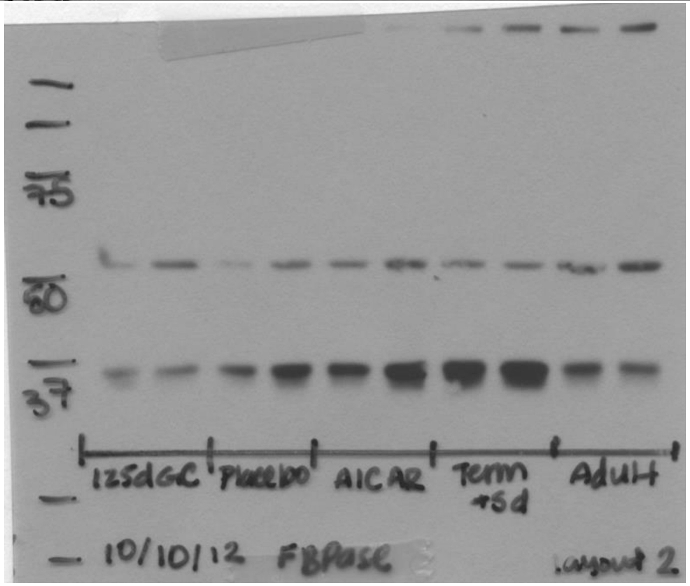

FBP1, Figure 6D

Placebo

AICAR

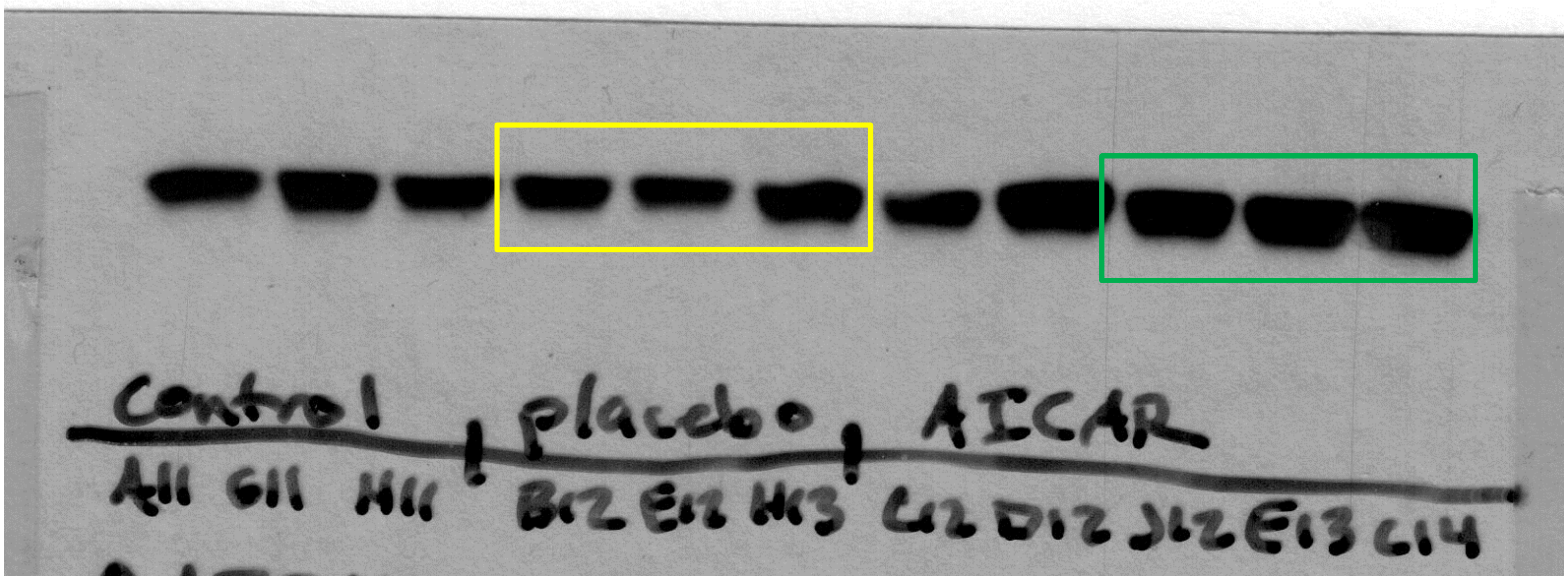

FBP1GAPDH Figure 6D

Placebo
AICAR

Acute AICAR

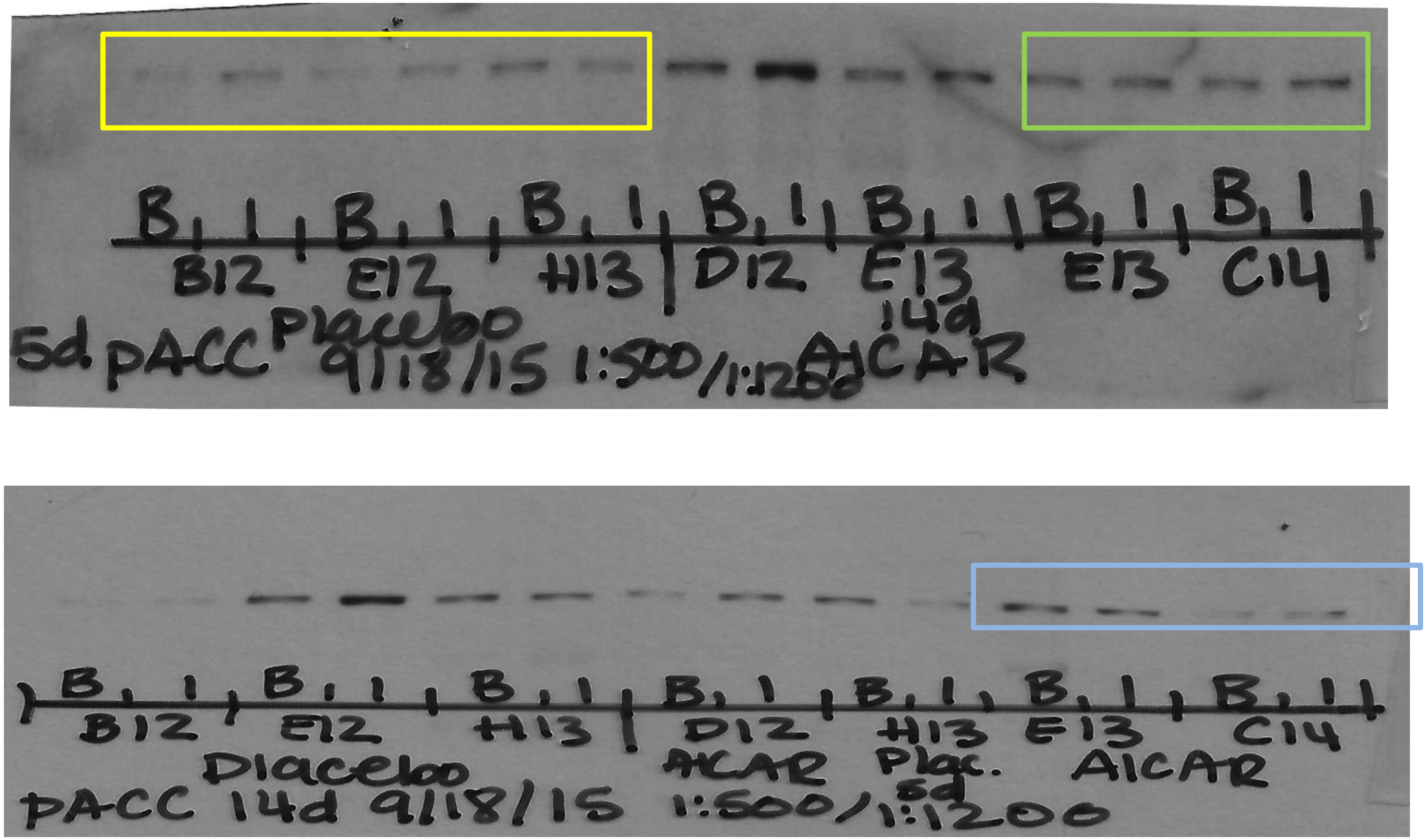

Placebo

Acute AICAR

Chronic AICAR

Figure 8, pACACA

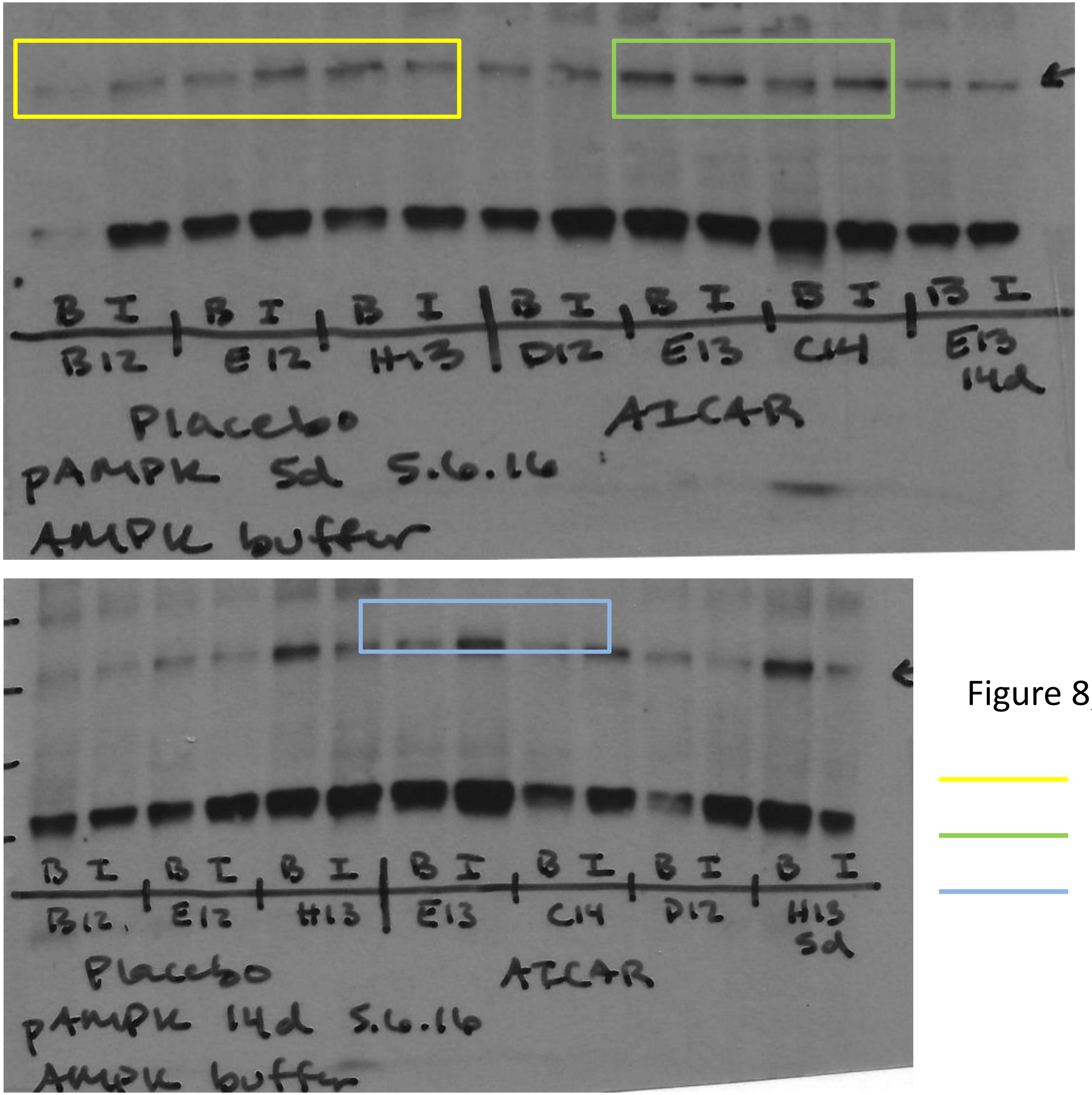

Figure 8, pPRKAA

Placebo

Acute AICAR

Chronic AICAR

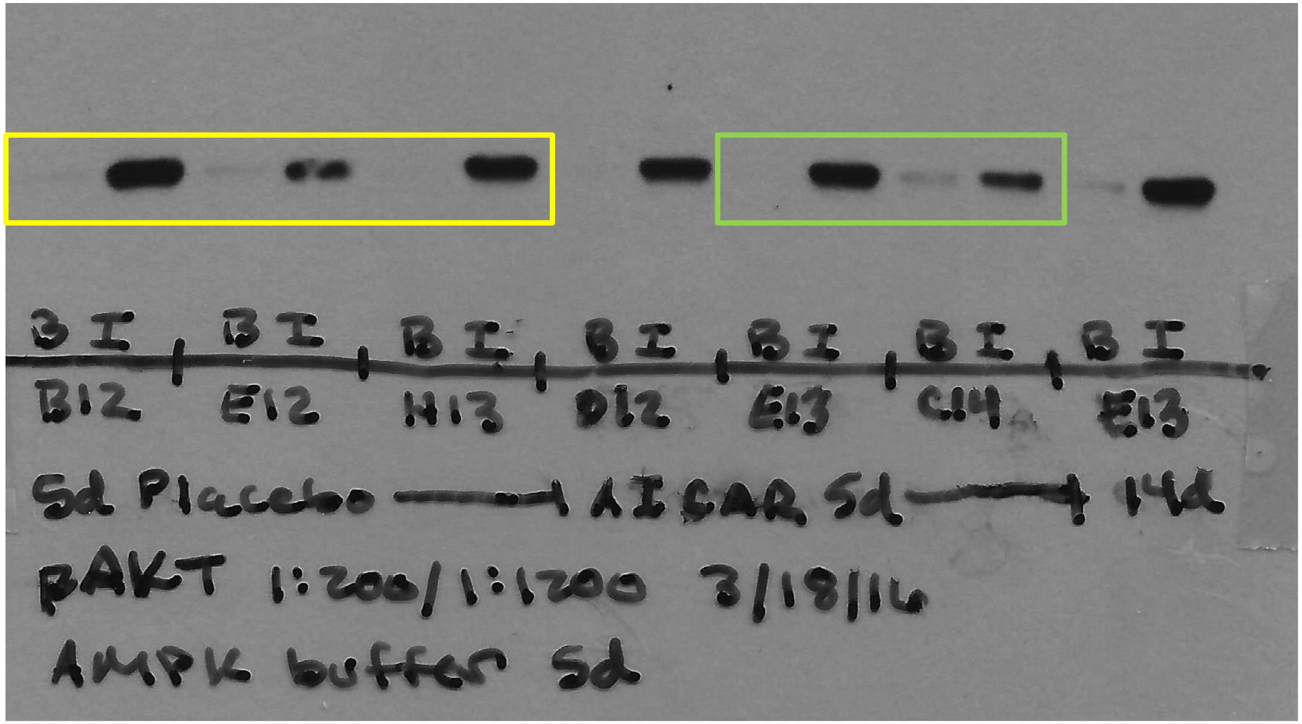

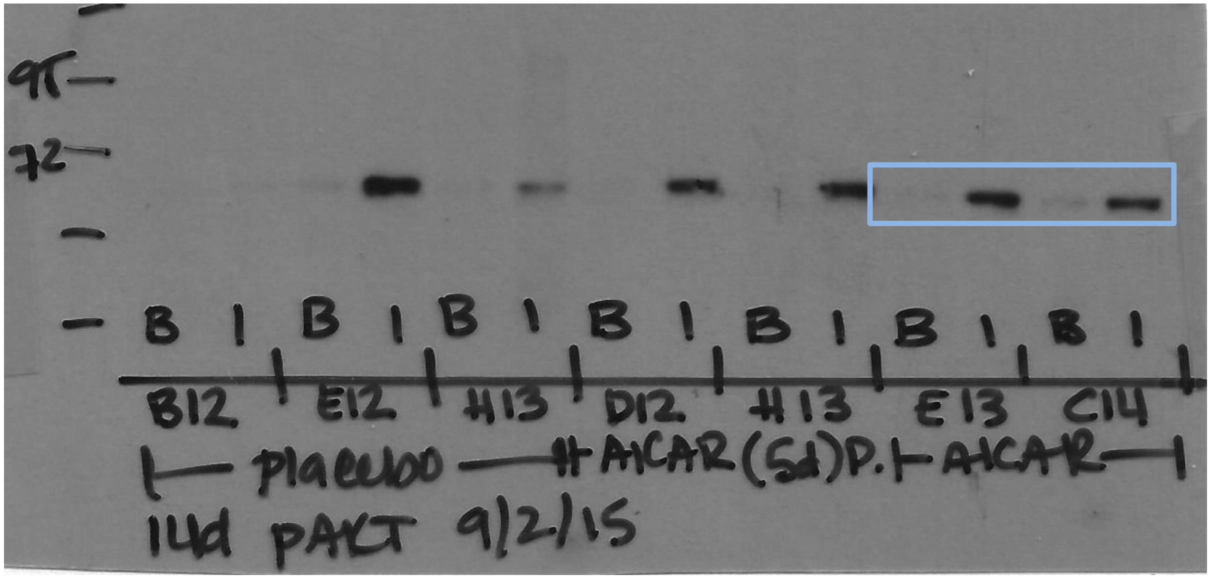

Figure 8, pAKT

Placebo

Acute AICAR

Chronic AICAR

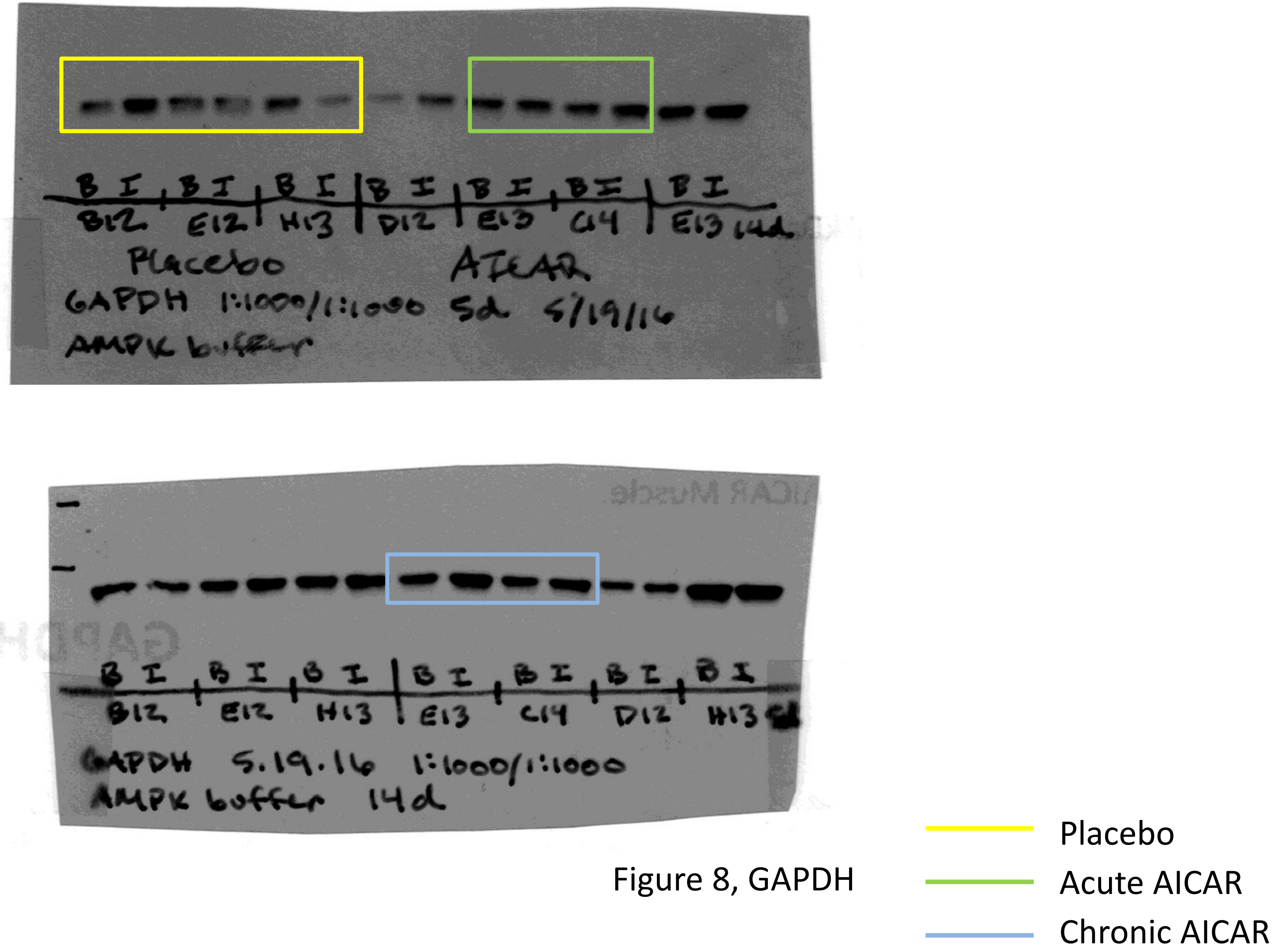

Figure 8, GAPDH
