# Supplementary figures and images for: Effects of intravenous AICAR (5-aminoimidazole-4-carboximide riboside) administration on insulin signaling and resistance in premature baboons, Papio sp
Source: PLoS One. 2018 Dec 12;13(12):e0208757. doi: 10.1371/journal.pone.0208757 (PMC6291136; doi:10.1371/journal.pone.0208757)

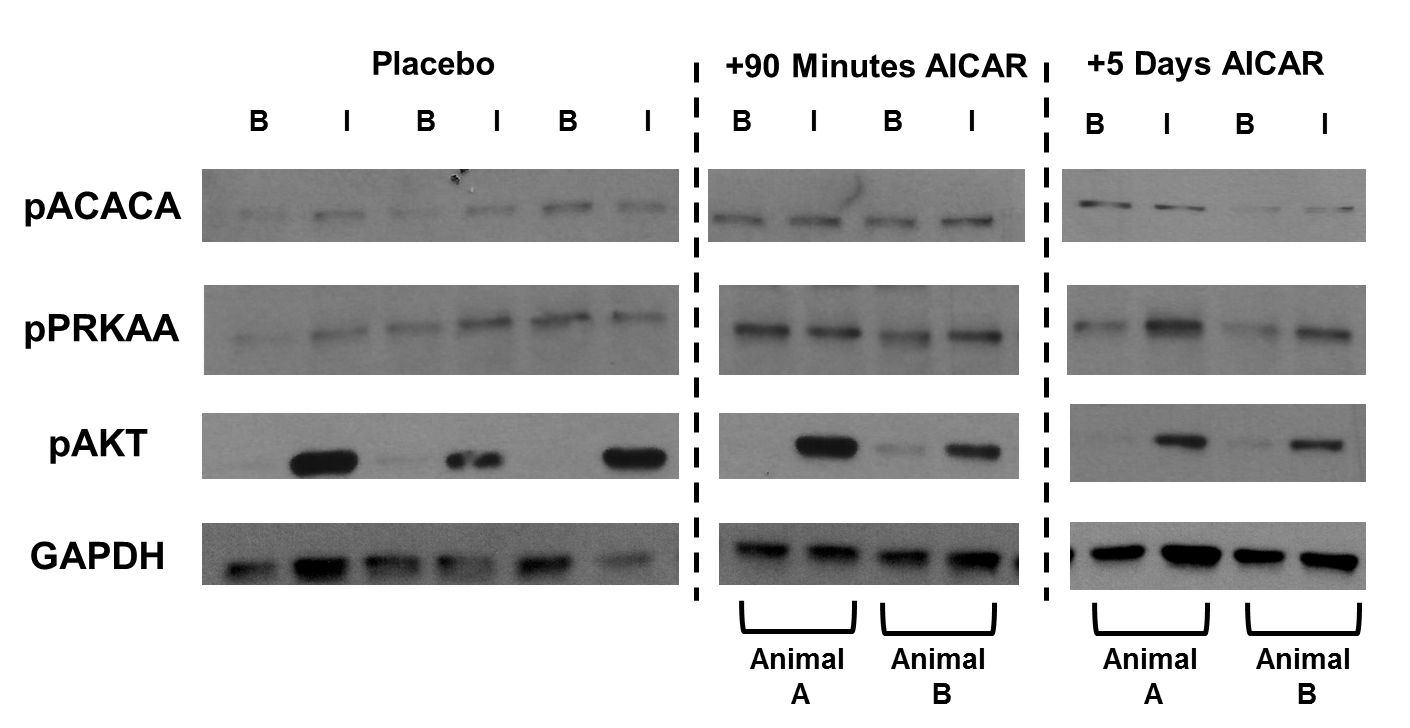

Supplement: S1 Fig — Western blot membranes demonstrating the effects of 90 minutes of AICAR infusion on skeletal muscle protein content of phosphorylated ACC, AMPK, and AKT are shown. B: baseline insulin levels (or clamp time +0 minutes), I: insulin-stimulated (or clamp time +120 minutes). A representative GAPDH loading control is also shown. (TIF) [file pone.0208757.s002.tif]
